# Irradiated microparticles suppress prostate cancer by tumor microenvironment reprogramming and ferroptosis

**DOI:** 10.1186/s12951-024-02496-3

**Published:** 2024-05-05

**Authors:** Zihan Deng, Binghui Li, Muyang Yang, Lisen Lu, Xiujuan Shi, Jonathan F. Lovell, Xiantao Zeng, Weidong Hu, Honglin Jin

**Affiliations:** 1 Department of Thoracic Surgery, ZhongNan Hospital of Wuhan University, Wuhan, Hubei China; 2 Center for Evidence-Based and Translational Medicine, Zhongnan Hospital of Wuhan University, Wuhan, China; 3 Department of Urology, Zhongnan Hospital of Wuhan University, Wuhan, China; 4 College of Biomedicine and Health and College of Life Science and Technology, Huazhong Agricultural University, Wuhan, 430070 China; 5 Department of Biomedical Engineering, University at Buffalo, State University of New York, Buffalo, NY 14260 USA

**Keywords:** Cancer immunotherapy, Microparticle, Ferroptosis, Immunogenic cell death, Tumor microenvironment, Macrophage reprogramming

## Abstract

Immunogenic cell death (ICD) plays a crucial role in triggering the antitumor immune response in the tumor microenvironment (TME). Recently, considerable attention has been dedicated to ferroptosis, a type of ICD that is induced by intracellular iron and has been demonstrated to change the immune desert status of the TME. However, among cancers that are characterized by an immune desert, such as prostate cancer, strategies for inducing high levels of ferroptosis remain limited. Radiated tumor cell-derived microparticles (RMPs) are radiotherapy mimetics that have been shown to activate the cGAS-STING pathway, induce tumor cell ferroptosis, and inhibit M2 macrophage polarization. RMPs can also act as carriers of agents with biocompatibility. In the present study, we designed a therapeutic system wherein the ferroptosis inducer RSL-3 was loaded into RMPs, which were tested in in vitro and in vivo prostate carcinoma models established using RM-1 cells. The apoptosis inducer CT20 peptide (CT20p) was also added to the RMPs to aggravate ferroptosis. Our results showed that RSL-3- and CT20p-loaded RMPs (RC@RMPs) led to ferroptosis and apoptosis of RM-1 cells. Moreover, CT20p had a synergistic effect on ferroptosis by promoting reactive oxygen species (ROS) production, lipid hydroperoxide production, and mitochondrial instability. RC@RMPs elevated dendritic cell (DC) expression of MHCII, CD80, and CD86 and facilitated M1 macrophage polarization. In a subcutaneously transplanted RM-1 tumor model in mice, RC@RMPs inhibited tumor growth and prolonged survival time via DC activation, macrophage reprogramming, enhancement of CD8^+^ T cell infiltration, and proinflammatory cytokine production in the tumor. Moreover, combination treatment with anti-PD-1 improved RM-1 tumor inhibition. This study provides a strategy for the synergistic enhancement of ferroptosis for prostate cancer immunotherapies.

**Graphical Abstract:**

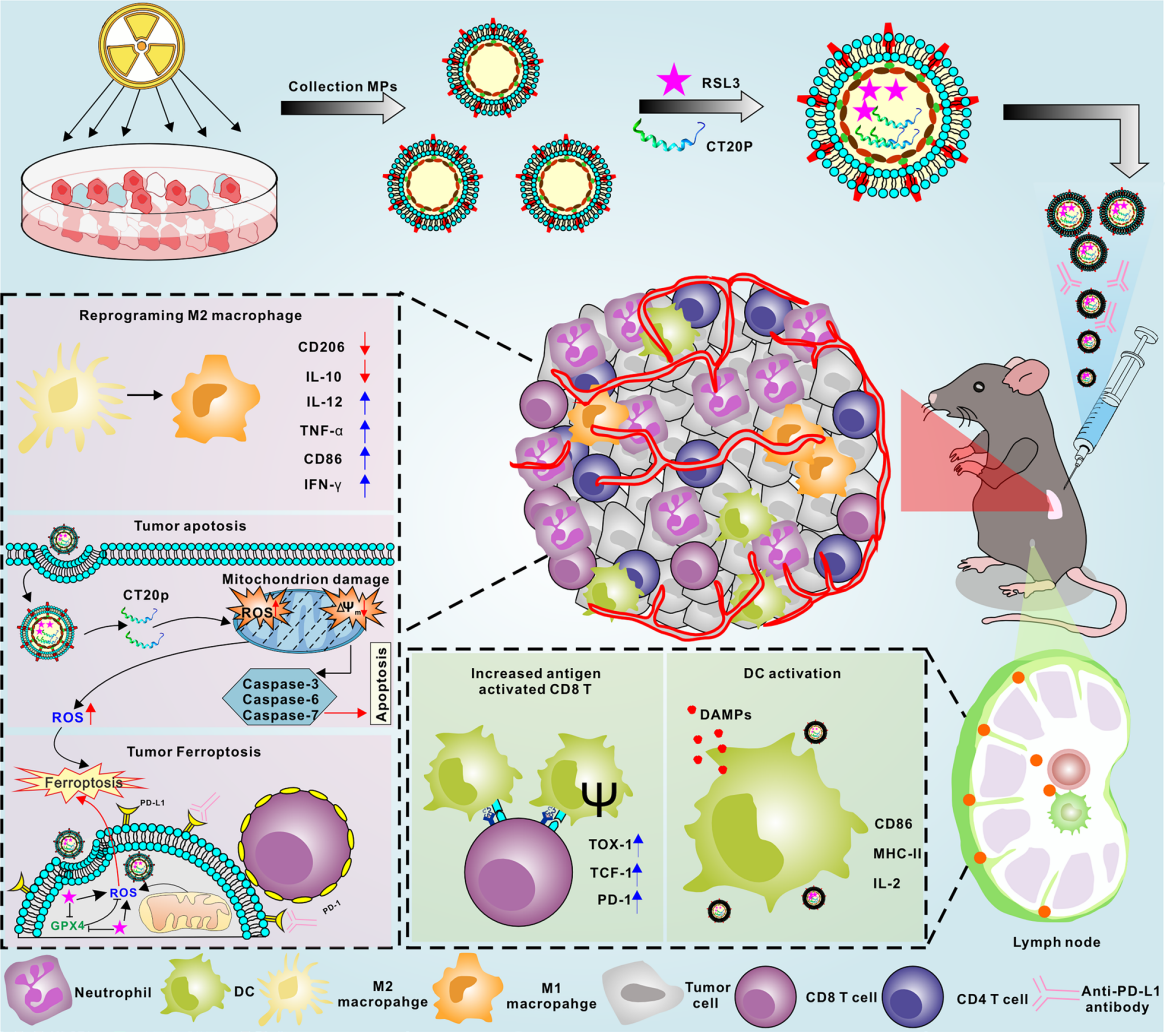

**Supplementary Information:**

The online version contains supplementary material available at 10.1186/s12951-024-02496-3.

## Introduction

Prostate cancer is an age-related tumor of the male genitourinary system that has a high incidence in the elderly population. Concurrent with the rapidly aging population, prostate cancer has become the leading male urinary system tumor in the world, and has one of fastest growing incidences among malignant tumors in males in the past decade [1]. The main treatment for prostate cancers involves prostatectomy combined with endocrine therapy and radiotherapy or chemotherapy. Unfortunately, after a period of treatment, most patients develop castration-resistant prostate cancer (CRPC) that is insensitive to treatment [2, 3]. However, as prostate cancer is normally considered a cold tumor, targeted therapy and immunotherapy often show only limited efficacy in the clinic [4, 5]. Therefore, there is an urgent need to explore the mechanism of the occurrence and development of prostate cancer and to develop new therapeutic drugs for prostate cancer.

Ferroptosis is a form of immunogenic cell death (ICD) that can enhance the ability of innate immune cells to recognize tumor cells and initiate the adaptive immune response [6]. Thus, ferroptosis can effectively promote the transformation of cold tumors into hot tumors, ultimately improving the response of cancer patients to immunotherapy [7–9]. SLC7A11 and GPX4 are two key molecules that inhibit ferroptosis, and are highly expressed in prostate cancer and CRPC [10]. Association studies have shown that nine genes associated with ferroptosis are closely related to the prognosis of patients with prostate cancer. Conversely, the CEMIP, HSPB1, and PANX2 genes, which interfere with the process of ferroptosis, can effectively promote the survival of prostate cancer cells, suggesting that ferroptosis-related genes may be prognostic biomarkers and potential drug targets for patients with prostate cancer [11–13]. Related studies have also suggested that the ferroptosis of neutrophils may promote the occurrence and development of tumors [14]; however, whether this phenomenon exists in prostate cancer requires further investigation. Moreover, the heterogeneity of prostate cancer frequently results in hyposusceptibility to ferroptosis, and the agents that trigger ferroptosis can also induce the death of T cells and non-tumor tissue damage, which limits the therapeutic effect of ferroptosis-inducing agents for cancer treatment [15]. Therefore, it is necessary to develop novel strategies to enhance ferroptosis with improved specificity to target tumor cells.

The tumor immunosuppressive microenvironment is the main cause of clinical prostate cancer recurrence and immunotherapy failure. The most common causes of tumor immunosuppressive microenvironment formation involve myeloid-derived suppressor cells (MDSCs), inhibitory neutrophils, regulatory dendritic cells (DCs), and tumor-promoting M2 macrophages [16–20]. It has been reported that commonly used radiotherapy techniques can improve the tumor microenvironment (TME) to some extent [21, 22]. However, radiotherapy treatment for prostate cancer commonly results in unavoidable adverse side effects, including lower urinary tract symptoms, intestinal complications, erectile dysfunction, and myelosuppression, which limits the application of radiotherapy in patients [23]. We previously reported that radiated tumor cell-derived microparticles (RMPs) are the main medium of the bystander effect induced by radiotherapy [24]. To some extent, the RMPs act as mimetics of radiotherapy, inducing tumor cell ferroptosis and the reprogramming of tumor-promoting M2 macrophages, which may activate type I interferon signaling through the cGAS-STING pathway [24]. As RMPs originate from the tumor tissue itself, they have an innate ability to target tumor cells. Furthermore, microparticles have been shown to be a good carrier of agents for cancer therapies [25, 26]. Our previous studies showed that RMPs loaded with agents and adjuvants can inhibit the progression of lung cancer and its brain metastasis [27–29], suggesting that RMPs derived from prostate cancer may a good drug carrier for the treatment of advanced prostate cancer.

To sum up, this study used RM-1 prostate cancer cells as a tumor model, and extracted the RMPs of the tumor cells to be repurposed as drug carriers that encapsulate RSL-3, a ferroptosis inducer targeting GPX-4 which is a key inhibitor of ferroptosis [30]. In addition, mitochondrial targeting peptide CT20p (peptide sequence: VTIFVAGVLTASLTIWKKMG, an inducer of apoptosis), which can induce multimodal death in tumor cells, was also loaded into RMPs. The CT20p is the C-terminal of the pro-apoptotic protein Bax, which can regulate the activity of Chaperonin-Containing TCP protein in prostate cancer, resulting in mitochondrial instability and cytoskeletal disruption to promote the effective killing of tumor cells [31]. The results of this study showed that RMPs encapsulating RSL-3 and CT20p (RC@RMPs) retain the characteristics of RMPs and effectively target and kill tumor cells in vitro and in vivo. As an apoptosis inducer, CT20p altered the mitochondrial membrane potential and aggravated ferroptosis by increasing the production of ROS and lipid hydroperoxide. Furthermore, RC@RMPs could activate DC cells and reprogram macrophage polarization. RC@RMPs enhanced both adaptive immunity via CD8^+^ T cells and innate immunity to effectively kill prostate cancer cells. Together, these data provide proof-of-concept for the use of RMP carriers in the treatment of prostate cancer.

## Methods

### Materials

The medium of cell culture was purchased from Gibco Life Technologies, Inc. (Grand Island, NY, USA), including Modified Eagle's Medium (DMEM), Roswell Park Memorial Institute (RPMI)-1640 medium. Fetal Bovine Serum (FBS) was obtained from Zhejiang Tianhang Biotechnology Co., Ltd. (Huzhou, China). Plasmocin was bought from InvivoGene (Toulouse, France) and penicillin/streptomycin was obtained from Biosharp (Hefei, China). Sterile 1 × phosphate buffered saline was purchased from Gibco Life Technologies, Inc. (Grand Island, NY, USA). All the cytokines were purchased from Biolegend (San Diego, CA, USA), containing granulocyte-macrophage colony-stimulating factor macrophage colony-stimulating factor, interleukin-4, interleukin-13, lipopolysaccharide and interferon-γ. RSL3 was bought from Selleck (Houston, TX, USA). CT20p and FITC-CT20p peptide was bought from BankPeptide Inc. (Hefei, China). Sucrose for electroporation buffer was gained from Sinopharm (China). Acetonitrile, methanol and chloroform for High Performance Liquid Chromatography (HPLC) were all purchased from Thermo Fisher Scientific (Waltham, MA, USA) and their purity was more than 99%. The reagents to detect the mode of cellular uptake** (**Chlorpromazine, 5-(N-ethyl-nisopropyl) amiloride, methyl-β-cyclodextrin, wortmannin and cytochalasin D) and lysosome escape (Lysotracker) were purchased from Yeasen (Shanghai, China). The fluorescence dye DiO, DiD and Rhodamine were obtained from Yeasen (Shanghai, China). H2DCFDA and PKH26 were bought from MedChemExpress (NJ, USA). FITC-Liperfluo was obtained from Dojindo (Japan). C11 BODIPY 581/591 and Phen Green SK diacetate were bought from GLPBIO (USA). Radioimmunoprecipitation assay buffer and the inhibitors of protease and phosphatase were obtained from Beyotime (Shanghai, China). For western blot, primary antibodies STING, p-STING, NFκB, p-NFκB, calreticulin and Laminin B1 were purchased from ABclonal* (*Boston, MA, USA) and GAPDH, CD63, CD81 and Alix were obtained from Proteintech Group, Inc. (Chicago, IL, USA). Secondary antibodies goat anti-mouse IgG H&L-HRP conjugated and goat anti-rabbit IgG H&L-HRP conjugated were bought from Abcam (Cambridge, UK). Collagenase IV and hyaluronidase were purchased from Biosharp (Hefei, China). All the antibodies for flow cytometry and immunocyte depletion were bought from Biolegend (San Diego, CA, USA). Clodronate liposomes were purchased from FormuMax (Silicon Valley, CA, USA). PD-1 mAb for treatment was obtained from BioXell (Italy).

### Cells culture

All the murine cell lines were purchased from China Center for Type Culture Collection (CCTCC, Wuhan, China), including prostate cancer cell line (RM-1), Lewis lung carcinoma cell (LLC), mammary cancer cell line (4T1), colon adenocarcinoma cell line (MC38), B16F10 melanoma cells, GL261 glioma cells, DC line (DC2.4) and monocyte cell line (RAW264.7). All the cell lines were treated with 25 µg mL^−1^ Plasmocin for at least two weeks and were mycoplasma^−^negative as determined by MycoProbe Mycoplasma Detection Kit (R&D Systems, Minneapolis, MN, USA). RM-1, 4T1 and LLC cells were cultured in DMEM while other cells were maintained in RPMI 1640 medium. Bone marrow-derived dendritic cells and macrophages (BMDCs & BMDMs) from C57BL/6 J mice were generated as previous descriptions in RPMI 1640 medium [32, 33]. All the mediums were added with 10% (v/v) FBS and 100 μg mL^−1^ penicillin/streptomycin.

### Preparation of RMPs

In 10 cm cell culture dishes, 6 × 10^6^ RM-1 cells were planted and irradiated with a single dose of 20 Gy by 6-MV X-rays (CHIRAD 225). Next, the medium of irradiated cells was renewed by 20 mL DMEM completed medium which its microparticles had been removed via centrifugation. 72 h later, the medium was collected, and cell debris were removed by 1000 g for 10 min and 14,000 g for 2 min. RMPs were gained from the supernatant via 14,000 g for further 60 min at 4 °C and washed with sterile 1 × PBS for 2 times. At last, the RMPs were resuspended with 1 × PBS for subsequent experiments.

### RMPs encapsulated with RSL-3 and CT20p via electroporation

RSL3 was dissolved in DMSO to a concentration of 10 mg mL^−1^. CT20 peptide (CT20p) was diluted by ultrapure water. RSL3 or CT20p was mixed with RMPs in a 1:2 ratio of mass in 400 mM sucrose solution. By an electroporation system (Gene Pulser X cell, Bio Rad, USA), 400 μL mixture was electroporated in 0.2 cm cuvettes via exponential pulse (voltage: 500 V; capacitance: 125 μF).

### Detection of RSL3 and CT20p in RMPs

The concentration of RSL3 in RMPs was measured by HPLC. HPLC analysis was conducted using a LC-2030C Plus instrument (Shimadzu, Japan). The separation was implemented with a ShimNex C18 chromatographic column (4.6 × 250 mm, 5 μm, 100 A, Shimadzu, Japan). Three times volume of acetonitrile was mixed with RMPs and then added chloroform (1:2, v/v). After vortexed, the mixture was centrifuged at 10,000 g for 5 min and the lower layer was extracted for measurement. As the standard solution, 10 mg RSL3 was dissolved in acetonitrile and chloroform in a ratio the same as RMPs. All the samples were filtered through a 0.45 μm polytetrafluoroethylene filter. The components were separated and eluted by mobile phase eluted (A: methanol; B: acetonitrile) in the column at 25 °C with a flow rate of 1 mL min^−1^. An ultraviolet wavelength (254 nm) was selected for the detection of RSL3. For CT20p detection, FITC-CT20p was capsuled into RMPs by electroporation in different conditions and relative fluorescence units (RFU) was measured via SpectraMax^®^ iD3 microplate reader (MOLECULAR DEVICES, CA, USA).

### Quantification of RMPs

The quantification of RMPs were determined by their protein concentrations. Radioimmunoprecipitation assay buffer were applied to lyse RMPs at 4 °C for half an hour. Then, the lysis was centrifuged at 12,000 g for 30 min at 4 °C and the supernatant was transferred into a new centrifuge tube for protein concentration measurement by BCA Protein Assay Kit (Thermo Fisher Scientific).

### Characterization of RMPs Size and Transmission electron microscopy (TEM) Determination

One milliliter of 30 ng mL^−1^ RMPs were taken for the measurement of the particle size and polydispersity index by Malvern laser particle size analyzer (Zetasizer Nano ZSP). For further identification of the sizes and morphology of RMPs were washed by ddH_2_O, deposited on copper mesh and then observed by TEM (HT7700-SS/FEI Tecnai G20 TWIN).

### Detection of drug release of RMPs

To simulate a biologically relevant environment, RMPs were incubated with mouse serum for different times at 37 °C. RMPs were collected and washed, and the concentration of RSL3 and CT20p in RMPs was detected by HPLC and microplate reader respectively.

### Cell viability measurement

All the cells to evaluate viability were planted into 96-well plates (5000 cells per well). After 24 h growing, distinct RMPs were treated with the cells for further 48 h. A cell counting kit-8 (CCK-8) assay kit (Meilunbio, Dalian, Chian) was used to measure cell viability.

### In vitro cellular uptake assay

To evaluate the cellular uptake of RMPs, different cell lines were planted into 6-well plates and incubated with DiO pre-dyed MPs for 3 or 6 h. The cells were collected, washed by PBS and analyzed through flow cytometry (Beckman CytoFLEX S, USA).

### Identification of modes of RMPs uptake and lysosome escape assay

To identify the ways of RMPs uptake by RM-1 tumor cells, Chlorpromazine (CPZ) (10 μg mL^−1^), 5-(N-ethyl-nisopropyl) amiloride (EIPA) (100 μM), methyl-β-cyclodextrin (MβCD) (5 mM), wortmannin (50 nM) and cytochalasin D (1 μM) were incubated with RM-1 cells for 2 h respectively. Subsequently, the DiO-labelled RC@RMPs were added for an incubation of 4 h and there cells were collected for mean fluorescence intensity (MFI) detection via flow cytometry. For clarifying the role of energy dependent phagocytosis in RMPs uptake, RM-1 cells were incubated with RMPs at 4 °C for 4 h. To measure lysosome escape of RMPs, RM-1 were incubated with DiO-labelled RC@RMPs for 24 h and dyed by Hoechst 33324 and Lysotracker. The merge of DiO and Lysotracker was evaluated as the mass of RC@RMPs in lysosome.

### Identification of the colocalization of CT20p and mitochondrion

RMPs with CT20p-Rhodamine B and Rhodamine B were added into the cells which were subsequently incubated with 100 nM MitoTracker Green® FM at 25 °C for 30 min. Confocal laser scanning microscopy (Carl Zeiss LSM710) was used to observe the colocalization of CT20p and MitoTracker Green^®^ FM (standing for mitochondrion) whose Pearson correlation coefficient was calculated by image J software.

### Analysis of cell apoptosis and ferroptosis

Cells were cultured in 24-well plates (30,000 cells per well) and then incubated with PBS (control), 5 μg mL^−1^ RSL3, 20 μg mL^−1^ different RMPs containing RMPs, RSL3@RMPs, CT20p@RMPs and RC@RMPs for 24 h. The addition of 1 μg mL^−1^ lipopolysaccharide (LPS) served as positive control of DC activation. The cells were harvested for relevant measurement. Apoptosis was evaluated by Annexin V-Alexa Fluor 488/7-AAD apoptosis detection kit (yeasen, China). The protocol complied with the instruction of the kit. To determine mitochondrial membrane potential, JC-1 assay kit (yeasen, China) was applied. The method to dye and measure cell apoptosis and mitochondrial membrane potential obeyed the instruction of the kits. To evaluate total ROS, lipid hydroperoxide, lipid ROS and Fe^2+^ level, the cells were respectively labelled with H2DCFDA (10 μM), FITC-Liperfluo (5 μM), C11B-BODIPY 581/591 (10 μM) and Phen Green SK diacetate (10 μM) in 1 mL PBS for 30 min at 37 °C in a cell culture incubator. Subsequently, these cells were washed with PBS twice and resuspended with 200 μL PBS and analyzed via flow cytometry.

### Western blotting

All the MPs and cells were lysed by RIPA buffer with the inhibitors of protease and phosphatase at 4 °C for 30 min, and then centrifuged at 12,000 g for 30 min at 4 °C. The mass of the sample loading was adjusted to the same according to their protein concentrations that were detected by BCA Protein Assay Kit. The samples were separated by SDS-PAGE and transferred to polyvinylidene difluoride membrane after boiled for 5 min. The membranes block by 5% not-fat milk at room temperature for 1 h and incubated with related primary antibodies at 4 °C overnight. With several wash by Tris-buffered saline with 0.05% Tween-20, secondary antibodies incubated with the membranes at room temperature for 1 h. NcmECL Ultra (P10100, NCM Biotech) was applied for chemiluminescent exposure of the blot.

### Mice

Male C57BL/6 J mice (aged 6–8 week, weighted 18–20 g) were purchased from SHULAIBAO Biotech. All mice were kept in micro-isolator cages, and the experimental protocols were approved by the Hubei Provincial Animal Care and Use Committee and were in compliance with the experimental guidelines of the Animal Experimentation Ethics Committee of Huazhong Agricultural University (No. HZAUMO-2024-0050).

### In vivo cellular internalization assay

To identify RMPs uptake by cells in tumor in vivo, we intratumorally injected 100 µL PKH26 marked RMPs to mice with RM-1 tumor burden. 24 h later, the mice were sacrificed and the tumors were digested into single cell for flow cytometry analysis before they were incubated with antibodies of CD45 (clone S18009F), CD3 (clone 17A2), B220 (clone RA3-6B2), CD11b (clone M1/70), Ly6G (clone S19018G), F480 (clone BM8), CD11c (clone N418), MHCII (clone M5/114.15.2) and NK1.1 (clone PK136). Besides, some tumor tissues were fixed, dehydrated and sectioned into frozen sections which were going to stain by related antibodies and observed via confocal laser scanning microscopy. To image the distribution of RMPs, RC@RMPs were stained with DiD, the 540/20 nm excitation filter and 620/20 nm emission filter were used and the exposure time was 15 s.

### Subcutaneously implanted prostate tumor model and treatment with RMPs

RM-1 tumor cells (1 × 10^6^ cells in 100 μL PBS) were subcutaneously implanted into right back. Five days after tumor inoculation, mice with uniform tumor volume were randomly divided into 7 groups including control group, PD-1 mAb group, RMPs group, RSL3@RMPs group, CT20p@RMPs group, RC@RMPs group and RC@RMPs combined with PD-1 mAb group, and were treated with corresponding RMPs (intratumoral injection with 100 µg in 100 µL PBS) and PD-1 mAb (10 mg/kg, intraperitoneal injection) at 6, 8, 10, 12 and 14 days after grouping. Vernier caliper was applied to measure the length (*L*) and width (*W*) of subcutaneous tumors every other day. The volume of tumor was calculated by the formula *V* = (*L* × *W*^*2*^)/2. Mice were sacrificed when the tumor volume reached 1000 mm^3^.

### Detection of immunocytes in tumor and draining lymph nodes (dLNs)

RM-1 tumors from mice were digested into single cell by cutting into small pieces and incubating with Collagenase IV (0.32 mg mL^−1^) and hyaluronidase (0.5 mg mL^−1^) for 1 h at 37 °C. The tumor cells were filtered through 70 μm cell strainer after lysis of RBCs. All the samples were blocked Fc receptors followed by incubating with detection antibodies containing CD3 (clone 17A2), CD8 (clone SK1), CD69 (clone H1.2F3), CD4 (clone GK1.5), PD1 (clone 29F.1A12), TOX (clone 6E6D03), TCF1 (clone 7F11A10), CD11c (clone N418), CD11b (clone M1/70), CD86 (clone GL-1), MHCII (clone M5/114.15.2), CD44 (clone IM7), CD62L (clone MEL-14) and Zombie NIR^™^, and then measured via flow cytometry.

### Cytokines detection

RM-1 tumors from mice were weighted and grinded into homogenate. The supernatant was collected by 6000 g centrifugation for 20 min at 4 °C. The LEGENDplex Mouse Cytokine Release Syndrome Panel (13‐plex) with VBottom Plate (purchased from Biolegend) was used for cytokine detection.

### Immune cell depletion

T helper cells and CTLs were depleted by CD4 (clone GK1.5) and CD8 (clone 2.43) antibodies respectively. One day before treatment, 200 μg antibodies were intraperitoneal injected into mice for 5 times at 2 day intervals. Macrophages were depleted by clodronate liposomes. One day before treatment, 200 μL clodronate liposomes were intravenously injected into mice for 5 times at 3 day intervals. Neutrophils were depleted by Ly6G (clone 1A8) antibody. One day before treatment, 200 μg antibodies were intraperitoneal injected into mice for 5 times at 2 day intervals.

### Histology, antibody staining, and imaging

To observe different immunocytes in tumor tissue, RM-1 tumors separated from mice were frozened in optimal cutting temperature (OCT) compound and sectioned followed by paraformaldehyde fixation. Then the slides were blocked by 1% BSA for 20 min at room temperature and incubated with antibodies of F4/80, CD206, CD3e and Ly6G for half an hour at 4 °C. After washed twice by PBS, 4,6-diamidino-2-phenylindole (DAPI) were added with the slides for nucleus. For toxicity assessment, the separated RM-1 tumors were fixed in 4% paraformaldehyde overnight, dehydrated and embedded in paraffin. The tissues were sectioned and stained by hematoxylin-eosin.

### Statistical analysis

All the data were analyzed by Prism software (GraphPad Prism 6.0 software). The log-rank (Mantel-Cox) test was applied to compare survival rates between groups. Kaplan-Meier analysis was used to analyze tumor growth, and comparisons of three or more groups were calculated by one-way analysis of variance (ANOVA). Two-tailed unpaired t test or the Mann-Whitney U test was performed to determine the significance of two groups. *P* values of < 0.05 were determined statistically significant. Data are presented as means ± SEM. *P < 0.05; **P < 0.01, ***P < 0.001, ****P < 0.0001 and ns stands for no significant.

## Results

### Prostate cancer gene expression patterns and immunological correlation of ferroptosis pathway factors

**Fig. 1:**
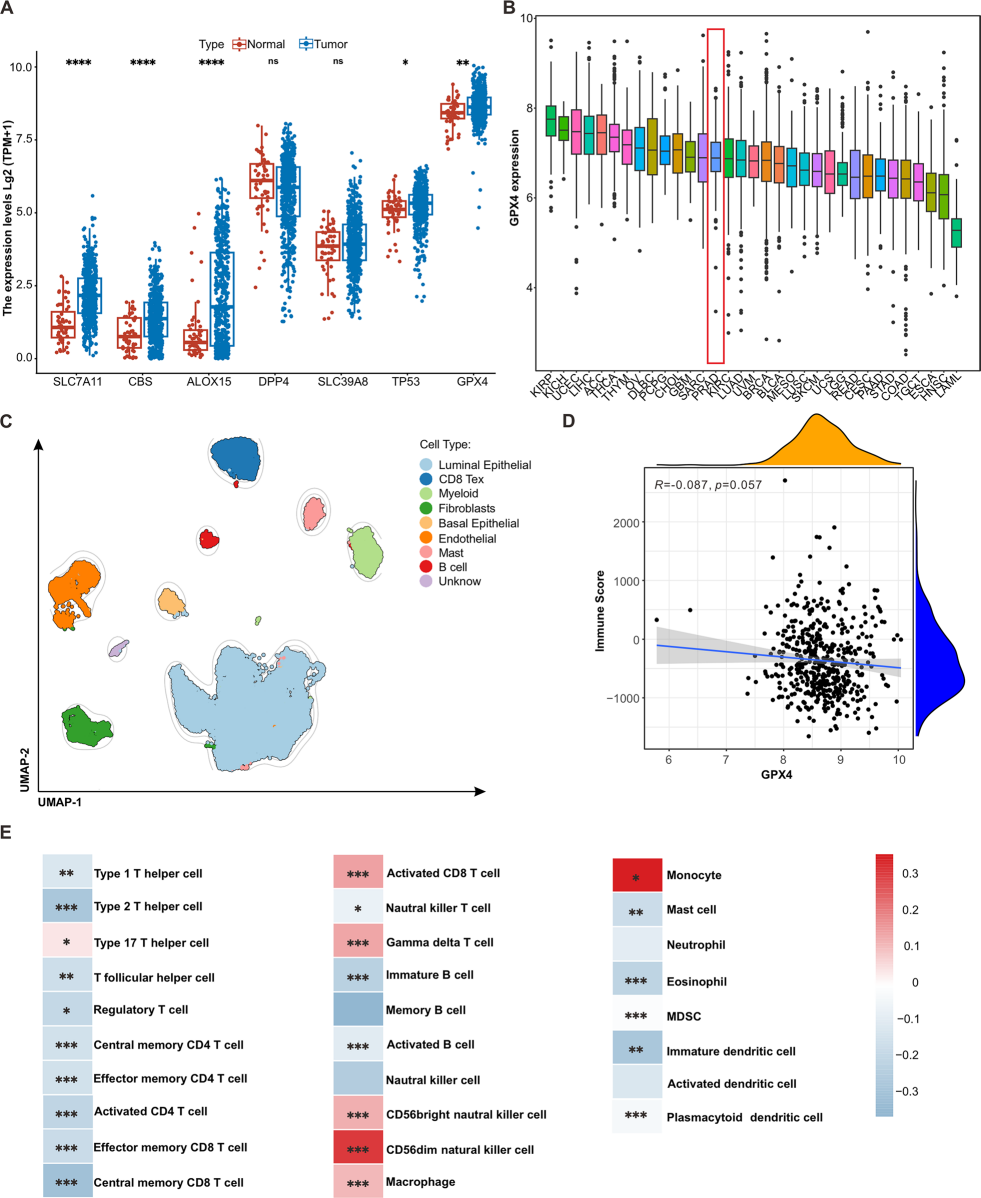
Key factors of ferroptosis significantly increased in tumor tissue and correlated with infiltration of different immune cells in patients with prostate cancer. **A** Difference of ferroptosis pathway factors expression between carcinoma and paracancerous tissue of prostate cancer. **B** The expression of GPX4 in pan-cancers. **C** The clusters of single cell transcriptome analysis of immunocytes in prostate cancer. **D** Correlation between GPX4 expression and immune score in prostate cancer. **E** Correlation between ferroptosis pathway factors and distinct immune cells in microenvironment of prostate cancer. Spearman correlation analysis was used to determine significant differences by P-value. *P < 0.05, **P < 0.01, and ***P < 0.001

To investigate the relationship between ferroptosis-related genes and prostate cancer, we analyzed data for 88 ferroptosis-related genes from published dataset [34] to identify differentially expressed genes in prostate cancer versus normal samples (|log2FC|> = 1, p < 0.05). We obtained a total of 8 ferroptosis-related genes (“SLC7A11”, “CBS”, “ALOX15”, “DPP4”, “SLC39A8”, “TP53”, and “GPX4”) in prostate cancer and paired normal samples. GPX4 showed significantly higher expression in prostate cancer (*P* < 0.05) and was associated with infiltrating immunocytes (Fig. 1A). We further explored the clinical relationship between GPX4 and prostate cancer using The Cancer Genome Atlas (TCGA) database. We investigated the expression of the GPX4 gene in different cancers, and found that GPX4 was highly expressed in the majority of moderate cancers, including prostate cancer (Fig. 1B). The TME of prostate cancer is relatively devoid of immune infiltration compared to other malignancies. Therefore, we reanalyzed published single-cell data on prostate cancer to explore the infiltration of immune cells in prostate cancer. The vast majority of cells in prostate cancer were epithelial cells, and the T cells present were predominantly were exhausted CD8^+^ T cells, confirming the immune desert phenotype of prostate cancer (Fig. 1C). 28 immune cell infiltration scores were evaluated in prostate cancer according to single sample gene set enrichment analysis (ssGSEA), and the correlations between GPX4 expression and immune cell infiltration scores were calculated. There was no significant correlation between GPX4 expression and total immune infiltration scores (Fig. 1D). However, the infiltration of many important immune effector cells, such as activated CD8^+^ T cells and gamma delta T cells, have strong correlations with GPX4 expression in prostate cancer (Fig. 1E), indicating that GPX4 may be involved in shaping the tumor immune microenvironment. In summary, the ferroptosis pathway factor GPX4 is highly expressed in prostate cancer and was found to correlate with the infiltration of important immune effector cells. Hence, GPX4 may be a potential target for inducing ferroptosis to improve the immune desert status in prostate cancer.

### Preparation and characterization of RC@RMPs

**Fig. 2:**
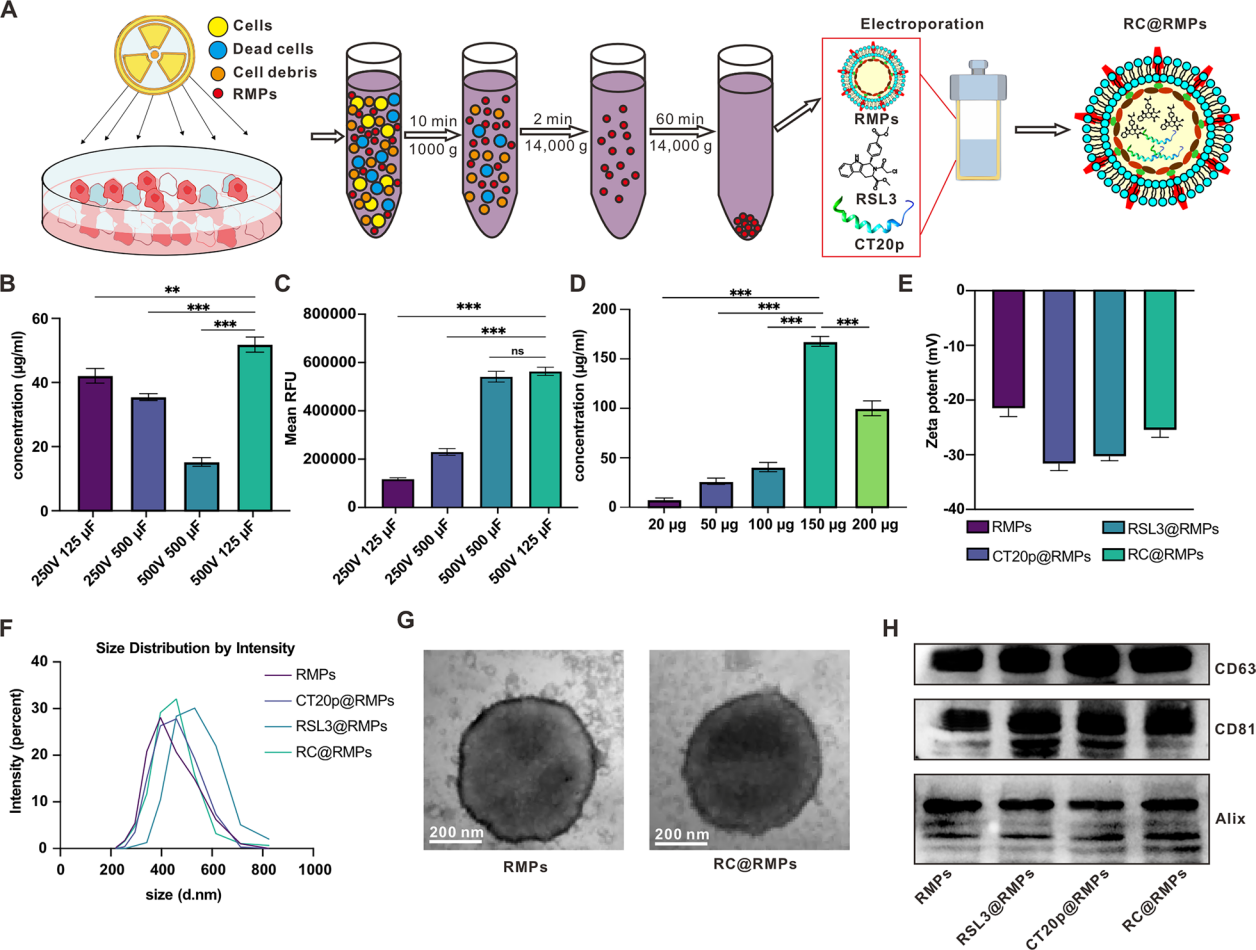
Preparation and characterization of RC@RMPs. **A** Schematic of RC@RMP preparation. Determination of electroporation conditions load RSL3 (**B**) or CT20p (**C**) in RMPs. 100 μg RSL3 or CT20p was mixed with 100 μg RMPs in the system. The most appropriate ratio of RSL3 (**D**) to RMPs was measured for loading most RSL3 in RMPs. The mass of RMPs in the system were set to 100 μg, and RSL3 concentration was detected via HPLC. **E** Zeta potential and **F** size of RMPs, RSL3@ RMPs, CT20p@RMPs and RC@RMPs measured by Malvern laser particle size analyzer. (G) TEM image of RMPs and RC@RMPs. **H** The expression of CD63, CD81 and Alix of RMPs, RSL3@ RMPs, CT20p@RMPs and RC@RMPs analyzed by Western blot. P-values were calculated by one-way analysis of variance (ANOVA). **P < 0.01 and ***P < 0.001

GPX4 is a peroxidase involved lipid metabolism that is vital for inhibiting ferroptosis [35]. GPX4 is correlated with the infiltration of various immune cells in prostate cancer, as indicated by the above bioinformatics analysis. The inhibition of GPX4 is considered a potential strategy for initiating ferroptosis [36]. RMPs, which are derived from radiotherapy-treated cells, are carriers of large quantities of DAMPs and have been shown to induce tumor cell death via ferroptosis [24]. We loaded the GPX-4 inhibitor RSL3 into RMPs to investigate the potential for a synergistic effect of GPX-4 inhibition and RMPs on ferroptosis to treat prostate cancer. Additionally, the C-terminal of the pro-apoptotic protein Bax, the CT20p, which induces mitochondrial damage, was also added into RMPs to intensify ICD for an enhanced anti-tumor immune response. The combined system (RC@RMPs) was constructed by obtaining and centrifuging the supernatant of irradiated RM-1 tumor cells, which had beed loaded with CT20p and RSL3 through electroporation. These methods are described in the experimental section and Fig. 2A. As the quantity of active agents in RMPs differed based on electroporation parameters and the ratio of RMPs to agents, we tried several different conditions for electroporation. Based on high-performance liquid chromatography (HPLC) analysis, the highest quantity of RSL3 in RMPs was achieved using following electroporation parameters: 500 V voltage, 125μF capacitance, and exponential decay wave mode, when the mass ratio of RSL3 to RMPs was 1:1 (Fig. 2B and Additional file 1: Figure S1). With the same electroporation parameters, most CT20p was encapsulated in RMPs (Fig. 2C). Maintaining the above electroporation conditions, we further found that 2:3 and 1:1 were the optimized mass ratios of RSL3 or CT20p to RMPs, respectively (Fig. 2D and Additional file 1:S2). Characterization of zeta potential (Fig. 2E) and size (Fig. 2F) showed no significant differences among RMPs, RSL3@RMPs, CT20p@RMPs, and RC@RMPs. Transmission electron microscopy (TEM) indicated that RMPs and RC@RMPs had a regular spherical morphology (Fig. 2G). Therefore, the loading of RSL3 and CT20p agents did not influence the structure of the RMPs. Western blot analysis demonstrated all the RMPs were rich in extracellular vesicle-associated proteins such as CD63 and CD81, whose expression was not influenced by encapsulation of CT20p and RSL3 (Fig. 2H). Thus, the RMPs that we extracted had the typical characteristics of extracellular vesicles. To assess RSL3 release from RC@RMPs in a physiological environment, RC@RMPs were incubated with mouse serum for different times at 37 °C. After 2 h of co-incubation, the concentration of RSL3 began to decrease and, by 72 h, had reached approximately 63.5% of the original concentration (Additional file 1: Figure S3A). Similarly, the release of CT20p (conjugated with FITC) loaded in RC@RMPs was measured by an enzyme-linked immunosorbent assay (ELISA). Twelve hours after co-incubation, the concentration of CT20p significantly decreased and reached approximately 72.6% of the initial concentration at 72 h (Additional file 1: Figure S3B). These results indicated that RC@RMPs can slowly release RSL3 and CT20p.

### The combination of RSL3 and CT20p in RC@RMPs synergistically induce ferroptosis in RM-1 cells

**Fig. 3:**
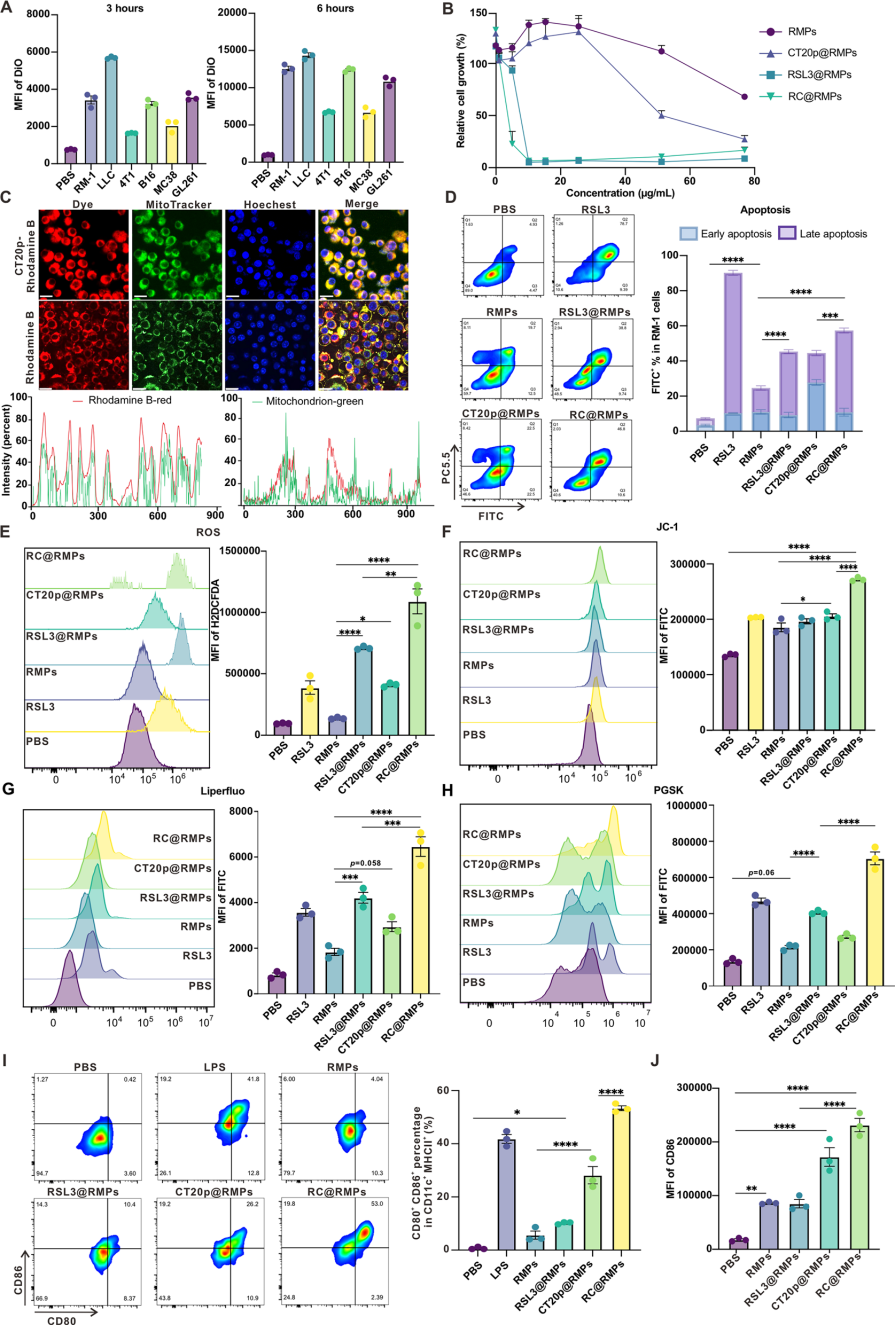
RC@RMPs induce ferroptosis of RM-1 tumor cell, activate DCs and promote M1 macrophages polarization. **A** The ability of different tumor cells to ingest RMPs. RM-1, LLC, 4T1, B16F10, MC38 and GL261 tumor cells were detected mean fluorescence intensity (MFI) in FL1 channel 3 h and 6 h after incubated. **B** Relative cell growth of RM-1 cells respectively incubating RMPs, RSL3@ RMPs, CT20p@RMPs and RC@RMPs for 24 h. (C) The colocalization of CT20p and mitochondrion through Confocal laser scanning microscopy, Scale bar: 20 μm. 100 μg CT20p-Rhodamine B was capsulated into 100 μg RMPs by electroporation. As control, Rhodamine B with equal amount of substance as CT20p-Rhodamine B was loaded into 100 μg RMPs. 20 μg mL^−1^ CT20p-Rhodamine B-RMPs and Rhodamine B-RMPs were added into the cells was observed via Confocal microscopy and Pearson correlation coefficient was calculated by image J software. **D** The rates of RM-1 apoptosis induced by different agents and RMPs. RM-1 were respectively incubated with PBS (control), 5 μg mL^−1^ RSL3, 20 μg mL^−1^ different RMPs containing RMPs, RSL3@RMPs, CT20p@RMPs and RC@RMPs. Annexin V-Alexa Fluor 488/7-AAD apoptosis detection kit were used to evaluate the percentages of apoptosis RM-1 24 h after different agents and RMPs incubation. Annexin V^+^ and 7-AAD^−^ stands for early apoptosis, while the cells with double positive are considered as late apoptosis. **E** ROS levels detected through H2DCFDA probe quantified by flow cytometry. The RM-1 cells in (**D**) were dyed with H2DCFDA which can react with ROS and emit fluorescence around 520 nm. **F** The evaluation of mitochondrial membrane potential of RM-1 cells in (**D**) with JC-1 assay kit. The wavelength of JC-1 emission changes from 590 to 529 nm when mitochondrial membrane potential decreases. The MFI of FL1 channel reflects mitochondrion damage degree. **G** FITC-Liperfluo (5 μM) and **H** Phen Green SK diacetate (PGSK) (10 μM) were incubated with the cells in (**D**) and measured MFI of FITC. **I** The percentage of CD80 and CD86 in CD11c^+^ and MHCII^+^ of DC2.4 cells and **J** CD86 of RAW264.7 incubating RMPs, RSL3@ RMPs, CT20p@RMPs and RC@RMPs for 24 h. The group incubated with LPS was positive control of DC activation. P-values were calculated by one-way analysis of variance (ANOVA). *P < 0.05, **P < 0.01, ***P < 0.001 and ****P < 0.0001

To identify whether RMPs can be ingested by tumor cells, 20 μg mL^−1^ DiO pre-labelled RMPs were incubated with some murine tumor cell lines for 3 or 6 h. We detected a high level of DiO fluorescence intensity in RM-1 cells, indicating that RM-1 cells can effectively take up RMPs (Fig. 3A). RC@RMPs uptake into RM-1 cells mainly occurred through clathrin-mediated endocytosis, macropinocytosis, caveolin-mediated endocytosis (CVME), actin polymerization-mediated phagocytosis, and energy-dependent endocytosis (Additional file 1: Figure S4A). Moreover, we observed that RM-1 cells effectively phagocytized DiO-labelled RMPs and most of the DiO signal was not co-localized with Lysotracker dye, suggesting that the RMPs could undergo lysosomal escape in RM-1 cells (Additional file 1: Figure S4B). Next, the toxicity of different RMPs to RM-1 tumor cells was evaluated. RC@RMPs were the most effective at eliminating RM-1 cells with the lowest IC (50) values (4.47 μg mL^−1^ vs 53.13 μg mL^−1^ for RMPs, 5.47 μg mL^−1^ for RSL3@RMPs and 47.43 μg mL^−1^ for CT20p@RMPs) (Fig. 3B). RSL3 is considered to induce ferroptosis by increasing ROS production and mitochondrial dysfunction. CT20p were added into RMPs to synergistically promote ferroptosis by mitochondrial damage. RMPs with CT20p-Rhodamine B and Rhodamine B (control) were incubated with RM-1 to identify the subcellular localization of CT20p. Confocal microscopy showed that CT20p-Rhodamine B colocalized with mitochondria as identified by staining with MitoTracker Green^®^ FM, which was confirmed by Pearson correlation coefficient analysis (Fig. 3C). Therefore, RMPs loaded with CT20p were targeted to the mitochondria. Flow cytometry analysis revealed that RC@RMP treatment resulted in a much higher rate of total apoptosis in RM-1 cells compared to the other groups (Fig. 3D). We found that RSL3 in large part caused late apoptosis, which was difficult for tumor cells to recover from; CT20p was able to induce early apoptosis by producing damaged mitochondria, though this could be reversed by mitophagy (Fig. 3D). The expression of calreticulin was upregulated with RMP treatment, including all the drug-loaded RMPs, suggesting that our treatments induced ICD (Additional file 1: Figure S5). Mechanistically, the toxicity of RC@RMPs was primarily related to the induction of ferroptosis, as indicated by increased total ROS production, increased lipid peroxidation, increased Fe^2+^ levels, and decreased mitochondrial membrane potential, which occurred to a greater extent in cells treated with RC@RMPs compared to other RMPs (Fig. 3E–H). ROS measurements based on H2DCFDA mean fluorescence intensity (MFI) were significantly higher in cells treated with RSL3@RMPs or CT20p@RMPs compared to RMPs, suggesting that both RSL3 and CT20p were able to elevate ROS production. RC@RMP-treated cells showed the highest ROS levels, demonstrating a synergism of RSL3 and CT20p (Fig. 3E). Meanwhile, there was greater green fluorescence in CT20p@RMP-treated cells compared with the RMPs group, confirming that CT20p played a key role in effects on mitochondria (Fig. 3F). To evaluate the effects of these treatments on ferroptosis, the levels of lipid peroxidation, lipid ROS, and iron ions were measured in RM-1 cells using FITC-Liperfluo, CD11-BODIPY, and Phen Green SK diacetate, respectively. RC@RMP-treated cells showed higher levels of all the ferroptosis indexes compared to RMPs, RSL3@RMPs, and CT20p@RMPs (Fig. 3G, Additional file 1: figure S6 and 3H). Overall, the combination of RSL3 and CT20p appears to synergistically trigger ferroptosis and may augment the subsequent immune response.

### RC@RMPs activate DCs and regulate macrophage polarization

As Antigen Presenting Cells (APCs), DCs are vital for initiating anti-tumor immunity. In the TME, they capture and process tumor antigens then present these antigens to tumor-specific T cells, which undergo clonal expansion, then recognize and eliminate tumor cells [37]. Macrophages are also APCs and function to activate T cells. However, tumor-associated macrophages (TAMs) always act as promoters of tumor progression by secreting anti-inflammatory cytokines such as IL-10 and TGF-β [20, 38]. Therefore, we measured the direct influence of RC@RMPs on APCs to evaluate the effect that RC@RMPs may have on reshaping the immunological environment of the tumor. DiO pre-labelled RMPs and RC@RMPs at different concentrations were incubated with DC2.4 and RAW264.7 cells. We found that the quantities of RMPs and RC@RMPs taken by APCs increased in a dose-dependent manner (Additional file 1: Figure S7). CCK8 cell toxicity assays showed that the cell growth of DCs treated with RMPs remained above 50% when the concentration of RMPs was less than 25 μg mL^−1^, indicating that DCs were insensitive to CT20p- and RSL3-loaded RMPs (Additional file 1: Figure S8A). Flow cytometry analysis showed that incubation with RC@RMPs could boost DCs activation by significantly increasing the expression of CD80 and CD86 in CD11c^+^ and MHCII^+^, compared to control groups and RMPs loaded with a single agent (Fig. 3I). Moreover, ROS production in both RSL3@RMP- and CT20p@RMP-treated cells was significantly higher than in cells given RMPs, and the combination of RSL3 and CT20p showed synergistic effects to activate innate immune pathways in DCs (Additional file 1: Figure S10). RMPs encapsulating DAMP-like DNA fragments generated by radiation may trigger the cGAS-STING pathway. Therefore, we performed Western blots to measure the expression of proteins related to cGAS-STING activation, including pSTING and pNF-κB (p65), in DC2.4 cells treated with different RMPs. The phosphorylation levels of STING and NF-κB were elevated when the cells were incubated with RMPs, RMPs loaded either RSL3 or CT20p, or RMPs loaded with both agents, compared to the control group (Additional file 1: Figure S11), indicating that RSL3 and CT20p loading does not influence RMP-induced activation of the cGAS-STING pathway. In contrast to DCs, RSL3-loaded RMPs showed relatively higher toxicity to macrophages (IC (50) values: 13.1 μg mL^−1^ for RSL3@RMPs and 9.08 μg mL^−1^ for RC@RMPs) (Additional file 1: Figure S8B). Macrophages treated with RC@RMPs expressed high levels of CD86, suggesting that RC@RMPs contributed to the M1 polarization of macrophages (Fig. 3J and Additional file 1: S9A). All RMPs reduced CD206 expression, with no significant differences among RMP-treated groups, demonstrating that RSL3 and CT20p loading maintains the ability of RMPs to inhibit macrophage polarization to the M2 state (Additional file 1: Figure S9B and S9C). Iron overload and ROS production are key factors that facilitate the induction of M1 macrophage polarization [39]. We found that macrophages treated with RC@RMPs had the highest levels of ROS and Fe^2+^ (Additional file 1: Figure S12), indicating that the effects of ferroptosis contribute to M1 polarization in the treatment. Together, these findings indicated that RC@RMPs can directly promote inflammation by activating DCs and promoting M1 macrophage polarization.

### RC@RMPs can be taken up by tumor cells and immunocytes in the TME in vivo

**Fig. 4:**
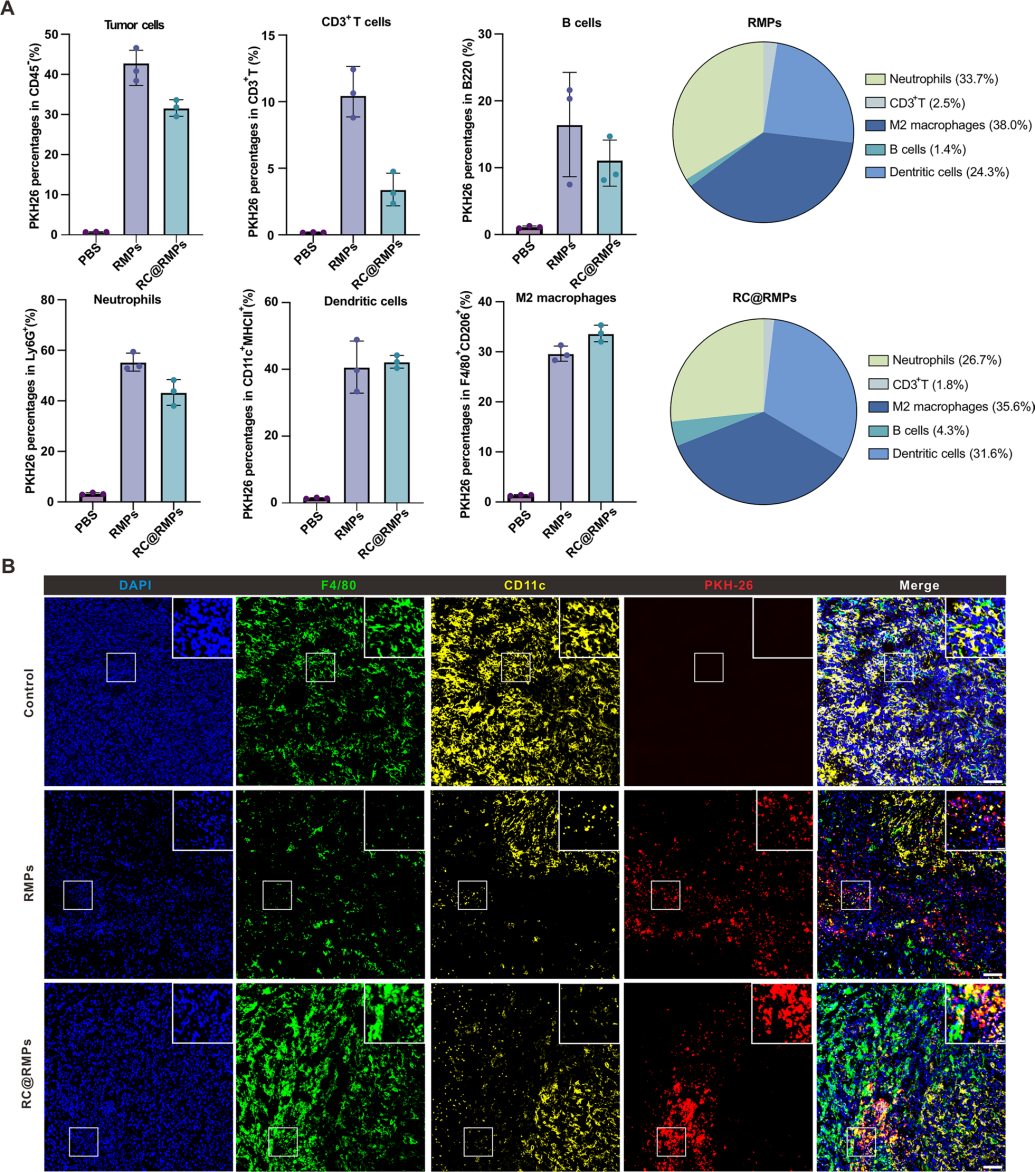
RC@RMPs accumulated in tumor cells and different subsets of immunocytes in tumor tissue in vivo. **A** Quantization of RMPs and RC@RMPs accumulation in tumor cells distinct immunocytes in tumor. 100 μg PKH26 pre-dyed RMPs and RC@RMPs were intratumorally injected and the mice were sacrificed 24 h after treatment. MFI of PKH26 was detected by flow cytometry and percentages of PKH26 positive immunocytes were calculated. **B** Representative immunofluorescence images showing RMPs internalization of F4/80^+^ macrophage and CD11c^+^ DCs in tumor tissue 24 h after treatment. Scale bar (original image): 200 μm, Scale bar (zoomed-in image): 50 μm

To explore the tissue distribution of RMPs in vivo, we performed intratumoral injections of 100 µg PKH26-labelled RMPs (100 µL) into mice previously implanted with RM-1 cells. 24 h later, the mice were sacrificed and the tumor and organs including the heart, liver, spleen, lung, kidney, brain, and dLNs were observed using the IVIS Spectrum Imaging System. The distribution of RMPs was limited to the tumor tissue, as shown in Additional file 1: Figure S13A. Then, we dispersed the tumor to generate a single cell suspension for flow cytometry analysis. There were no significant differences in the percentages of PKH26-positive tumor cells, T cells, B cells, neutrophils, DCs, or M2 macrophages between mice injected with RMPs and those given RC@RMPs (Fig. 4A and Additional file 1: S14). Tumor sections were stained using antibodies targeting neutrophils, DCs, and macrophages. The results confirmed that RMPs could be taken up by the relevant immune cells (Fig. 4B and Additional file 1: figure S13B).

### RC@RMPs reshape the tumor immune microenvironment and the combination of RC@RMPs and anti-PD-1 mAb shows synergistic antitumor activity

**Fig. 5:**
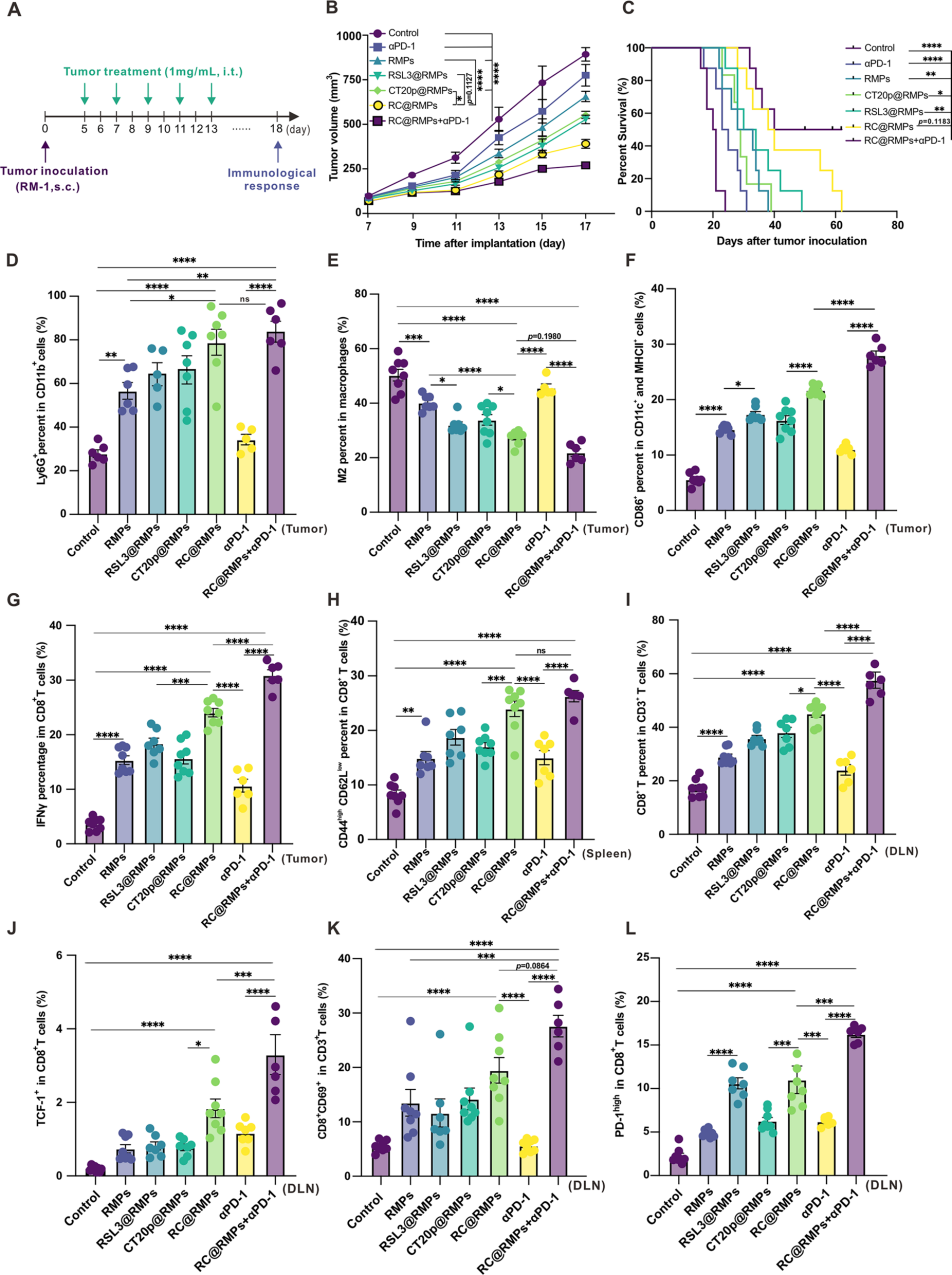
RC@RMPs treatment inhibited the growth of RM-1 tumor by remodeling infiltration of immunocytes, and the effect can be strengthened via combination with anti-PD-1 mAb. **A** Schematic of subcutaneously implanted RM-1 tumor treatment by intratumoral injection of RMPs, RSL3@ RMPs, CT20p@RMPs and RC@RMPs in a dose of 100 μg per mouse one time. Anti-PD-1 mAb was intraperitoneal injected (10 mg kg^−1^) in corresponding groups. **B** The curve of tumor growth by measuring tumor volumes every 2 days. **C** Survival curve of RM-1 burden mice in all groups. **D**–**L** The changes of immunocytes in tumor after treatment were analyzed by flow cytometry. The log-rank (Mantel-Cox) test was applied to compare survival rates between groups. P-values of the experiments were calculated by one-way analysis of variance (ANOVA). *P < 0.05, **P < 0.01, ***P < 0.001 and ****P < 0.0001

**Fig. 6:**
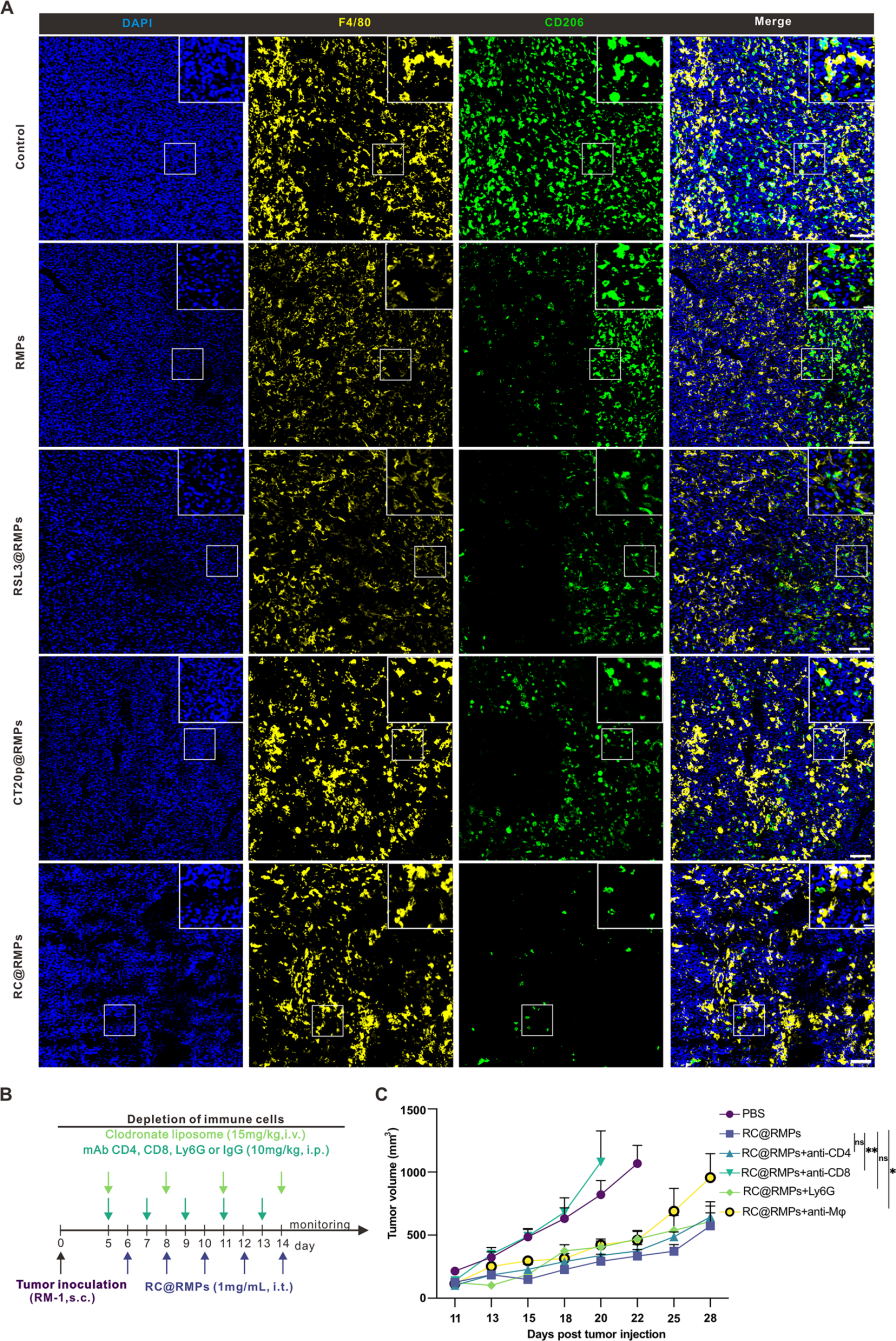
The anti-tumor effect of RC@RMPs depends on CD8^+^T cells and macrophages. **A** Representative immunofluorescence images showing the number of M2 macrophage in tumor tissue, scale bar (original image): 200 μm, Scale bar (zoomed-in image): 50 μm. **B** Schematic of subcutaneously implanted RM-1 tumor treatment by RC@RMPs with depletion of CD4^+^ T cells, CD8^+^ T cells, neutrophils and macrophages. **C** The curve of tumor growth by measuring tumor volumes of mice every 2 days. P-values were calculated by one-way analysis of variance (ANOVA). *P < 0.05, **P < 0.01, ns stands for no significance

In order to evaluate the therapeutic effect of RC@RMPs on prostate cancer, we treated subcutaneously implanted RM-1 tumors with control, RMPs, RSL3@RMPs, CT20p@RMPs, and RC@RMPs according to the treatment scheme shown in Fig. 5A. All treatments began at 7 days after RM-1 inoculation and the tumor sizes did not vary across the different groups (Additional file 1: Figure S15). RC@RMP therapy significantly reduced the growth of RM-1 tumors and prolonged survival time compared with mice treated with control, RMPs, RSL3@RMPs, or CT20p@RMPs (Fig. 5B-C). The median survival time of RC@RMP-treated mice was the longest (39 days) among all groups, although all mice reached ethical endpoint by 62 days after tumor inoculation (Fig. 5C). To clarify the effects of RC@RMP treatment on the immune microenvironment, immunocytes in the tumor were measured by flow cytometry (Additional file 1: Figure S16). We found a significant increase in the number of neutrophils, CD86^+^ MHCII^+^ DCs, total CD8^+^ T cells, IFN-γ^+^ CD8^+^ T cells, and memory CD8^+^ T cells, and a decrease in the number of M2 macrophages compared with the groups given control and RMPs (Fig. 5D–K and S17). The level of related cytokines were also measured, only IL-6 was found to be significantly elevated by RC@RMPs compared with the control and RMP groups (Additional file 1: Figure S18G). PD-1 is a negative regulator of the immune system that prevents overactivity and subsequent cytokine release syndrome. The expression of PD-1 is enhanced upon T cell activation, forming a negative feedback loop. Results showed that RC@RMPs significant promoted PD-1 expression, further indicating an uptick in T cell activity. However, RMPs also increased the expression of PD-L1 on macrophages, which can induce programmed cell death in T cells with high PD-1 expression. Therefore, we undertook a combined therapy strategy using an anti-PD-1 antibody and RC@RMPs to antagonize the immunosuppressive effect of the PD-1/PD-L1 pathway during RC@RMP treatment. The combination treatment promoted the inhibition of RM-1 growth (Fig. 5B) and resulted in a 50% survival rate 62 days after tumor inoculation (Fig. 5C). The combination therapy also significantly enhanced the presence of CD86^+^ MHCII^+^ DCs, the proportion of CD8^+^ T cells among CD3^+^ T cells, IFN-γ^+^ CD8^+^ T cells, and TCF-1^+^ CD8^+^ T cells, while decreasing the number of M2 macrophages, compared with monotherapy treatment with only RC@RMPs (Fig. 5D–L and Additional file 1: Figure S17). Furthermore, proinflammatory cytokines including CXCL9, TNF-α, CCL4, and CCL3 were also upregulated in the tumor following the combined therapy, further demonstrating the change in the immune environment (Additional file 1: Figure S18A–I). To identify the key immune subsets affected by our treatment, sections of tumor were stained for CD3, Ly6G, F4/80, and CD206 to observe the presence of T cells, neutrophils, and M2 macrophages. Histological analysis showed that RC@RMPs markedly increased T cell infiltration (Additional file 1: Figure S19A) and decreased the number of M2 macrophages present (Fig. 6A). To directly assess the role of infiltrating immunocytes, we depleted T helper cells, cytotoxic T cells, neutrophils, and macrophages using CD4 mAb, CD8 mAb, Ly6G mAb, and clodronate liposomes, respectively (Fig. 6B). These targeted cell subsets were rapidly depleted in peripheral blood within 24 h, which was confirmed by flow cytometry (Additional file 1: Figure S20). We observed that the depletion of CD8^+^ T cells and macrophages impaired the efficacy of RC@RMP treatment (Fig. 6C), suggesting that these two cell subsets are the main targets of RC@RMPs. Finally, we tested for possible toxicity following RC@RMP treatment. All mice in treated groups showed similar levels of alanine transaminase (ALT), aspartate transaminase (AST), blood urea nitrogen (BUN), creatinine (CREA), and complete blood count (CBC) indexes as the control group (Additional file 1: Figure S21A and Table 1). No abnormalities were observed in the heart, liver, spleen, lung, and kidney after the different RMP therapies, as determined by histopathological examination (Additional file 1: Figure S21B). Together, these results indicated that RC@RMPs successfully remodeled the immune desert environment of RM-1 tumors, and PD-1 blockade enhanced the effectiveness of this immune enhancement and tumor cell killing.

**Table 1:** Complete Blood Count (CBC) of the mice after treatment with RMPs

CBC/Group	Control	RMPs	RSL3@RMPs	CT20p@RMPs	RC@RMPs
WBC	7.7 ± 2.4	8.9 ± 1.6	5.8 ± 1.3	8.3 ± 2.6	7.8 ± 2.4
Lymph#	4.6 ± 1.5	5.6 ± 1.9	3.1 ± 1.4	6.7 ± 3.4	6.2 ± 1.9
Mon#	0.3 ± 0.2	0.3 ± 0.1	0.2 ± 0.1	0.3 ± 0.2	0.2 ± 0.1
Gran#	2.8 ± 0.7	3.0 ± 0.5	2.5 ± 0.6	3.1 ± 1.9	1.4 ± 0.5
RBC	7.9 ± 0.3	7.9 ± 0.4	7.8 ± 0.1	7.9 ± 0.8	9.1 ± 0.3
HGB	118.0 ± 3.6	113.3 ± 2.9	101.7 ± 17.1	118.0 ± 15.7	130.0 ± 6.2
HCT	38.0 ± 0.9	36.1 ± 0.9	32.6 ± 4.2	36.9 ± 4.9	41.2 ± 1.5
MCV	48.4 ± 1.2	45.7 ± 1.3	42.2 ± 3.8	46.4 ± 1.7	45.6 ± 0.5
MCH	14.9 ± 0.3	14.3 ± 0.4	13.0 ± 1.6	14.7 ± 0.5	14.3 ± 0.4
MCHC	310 ± 2.6	313.7 ± 1.2	310.0 ± 13.9	319.0 ± 0.1	314.7 ± 4.0
RDW	16.8 ± 0.5	16.5 ± 0.8	18.6 ± 2.5	16.2 ± 0.4	15.6 ± 0.4
PLT	1455.7 ± 131.51	1639.7 ± 212.9	1976.3 ± 266.1	1584.0 ± 267.4	1368.3 ± 282.7
MPV	5.2 ± 0.2	5.4 ± 0.4	5.4 ± 0.2	5.5 ± 0.5	5.3 ± 0.3
PDW	15.9 ± 0.1	15.9 ± 0.1	15.6 ± 0.2	15.8 ± 0.1	15.7 ± 0.1

## Discussion

Although ferroptosis is a potent trigger of the innate immune system, there remain significant obstacles for treatment strategies that focus on inducing ferroptosis. Many ferroptosis-inducing agents have limited efficacy against cold tumors due to their short half-life, hyposusceptibility of the tumor to ferroptosis, and toxicity to normal cells. Therefore, a major challenge for the clinical application of ferroptosis inducers is to determine strategies to improve the potency and tumor-targeting capabilities of ferroptosis-inducing agents. As one of the most crucial organelles in cell, mitochondria are rich in metabolism-related molecules which can trigger ICD [40]. Despite some controversy, targeting mitochondria to stimulate the release of DAMPs has been shown to cause ferroptosis via the release of ROS and free iron [41, 42]. Recent evidence indicates that radiation triggers ferroptosis and increases the susceptibility of cancer cells to ferroptosis [43, 44]. Mechanistically, radiotherapy impairs lipid metabolism through the promotion of ROS production and ACSL4 expression, and downregulation of SLC7A11, all of which mediate ferroptosis [45–47]. As radiotherapy derivatives, RMPs induce tumor cell ferroptosis and reprogram macrophage polarization through DAMPs generated by radiation, as previously demonstrated [24]. Furthermore, RMPs are microparticles that can act as carriers for active agents, with good stability and biocompatibility. As they are generated from tumor cells, RMPs are also able to target the tumor and act as a source of tumor antigens to APCs. Thus, the combination of RMPs and RSL3 may provide a new strategy to more effectively induce tumor cell ferroptosis and thus stimulate innate immunity.

In the study, we designed a combined system of RMPs loaded with the ferroptosis inducer RSL3 and apoptosis inducer CT20p (RC@RMPs). This system has some distinct advantages. (i) Firstly, the system demonstrates strong synergy to induce tumor cytotoxicity: RC@RMPs were significantly more effective at killing RM-1 cells through ferroptosis, compared to RMPs with a single agent (Fig. 3A–H). We first discovered that CT20p enhanced ferroptosis through the production of ROS, lipid peroxidation, and the disruption of the mitochondrial membrane potential (Fig. 3E–H). This peptide has been shown to target mitochondria and induce mitochondrial membrane hyperpolarization, which impairs the distribution and movement of mitochondria [31]. Consequently, mitochondrial metabolism is compromised, which results in ROS release and Fe^2+^ overload, accelerating the ferroptosis process [48]. Nevertheless, the complete mechanism of the synergy through which RSL3, CT20p, and RMPs promote ferroptosis requires further elucidation. We note that as we used relatively low doses of RMPs compared to previous literature [24], the RMPs alone did not initiate a significant ferroptosis effect, although DAMPs in the RMPs triggered innate immunity. (ii) Secondly, RC@RMPs caused DC activation and M1 macrophage polarization. ROS production is essential in tumor therapy, for it can not only increase ICD in tumor cells but also activate APCs. The highest expression levels of B7 molecules in DCs was achieved by RC@RMP treatment (Fig. 3I), which was associated with increased ROS levels that may have activated the *CD80/CD86* promoters via the release of Ca^2+^ and expression of positive transcription elongation factor b (P-TEFb) [49]. Moreover, DAMPs in RMPs can trigger the activation of the cGAS-STING pathway to facilitate the expression of type Ι interferon, and cause the autoactivation of APCs (Additional file 1: Figure S11). As our results have shown, all the RMPs promoted the phosphorylation of key components of the cGAS-STING pathway in DC2.4 cells (Additional file 1: Figure S11), suggesting that the synergistic activity of RSL3 and CT20p in RMPs were able to mobilize a more intense innate immune response. M1 macrophage polarization is essential in antitumor immunity, as demonstrated in Fig. 6C. Iron overload can promote glycolysis, ROS formation, p53 acetylation, inflammatory cytokine production, and consequent M1 polarization[39]. However, iron overload and ROS may not be the main factors that promote M1 macrophage polarization, as CD86 expression was not differentially expressed when comparing groups given RMPs and RSL3@RMPs, even though RSL3@RMPs produced much higher levels of iron and ROS than RMPs (Fig. 3J and Additional file 1: S12). Macrophages treated with CT20p@RMPs in vitro showed a stronger tendency towards M1 phenotypes, implying that CT20p facilitated M1 polarization (Fig. 3J), possibly by influencing other pathways of mitochondrial metabolism in macrophages. (iii) Thirdly, RC@RMPs were capable of targeting tumor sites without spreading throughout other organs, which is likely due to the nature of RMPs, which are derived from tumor cells. Our data confirmed that the RMPs did not distribute into other organs and tissues when injected intratumorally (Additional file 1: Figure S13A). Notably, the greatest RMP uptake was observed in macrophages within the tumor (Fig. 4A), indicating the importance of reprogramming macrophage polarization. The tumor-targeting of RMPs is critical for minimizing side effects by allowing appropriate dose reduction without compromising therapeutic effect. (iv) Fourthly, the combination therapy with anti-PD-1 mAb demonstrates a highly promising treatment modality. High PD-1 expression is a characteristic of activated CD8^+^ T cells and a vital checkpoint for immunosuppression by binding with PD-L1. RC@RMP treatment significantly enhanced the percentage of PD-1^+^ CD8^+^ T cells, which may limit the therapeutic effect. Addition of anti-PD-1 mAb to increase the presence of inflammatory immunocytes and cytokines in the TME successfully inhibited tumor growth and prolonged the survival time of mice burdened with RM-1 tumors. (v) Finally, RMPs show good biocompatibility and safety for use as cancer vaccines (Fig. 5). All our RMPs could be taken up by tumor cells through different cellular uptake mechanisms and escape from lysosomal degradation (Additional file 1: Figure S4). No obvious damage in the major organs was observed after treatment with RMPs (Additional file 1: Figure S21 and Table 1). (vi) Our strategy provides a drug delivery system that combines immune checkpoint therapy (ICT) and radiotherapy. RMPs are regarded as mimic of radiotherapy, as they carry inflammatory molecules and DAMPs generated from radiotherapy, which triggers ICD. Furthermore, to facilitate precision medicine, RMPs can encapsulate distinct agents for different targets, that providing a versatile platform for cancer treatment. To summarize, RC@RMPs can induce tumor cell ferroptosis through the synergistic action of RSL3 and CT20p, and remodel the tumor immune microenvironment of prostate cancer by DC activation and M1 macrophage polarization.

There remain some limitations in our system. The toxicity of the RC@RMPs was not entirely specific to prostate cancer cells, leading to the death of macrophages and limiting the inflammatory effects of M1 macrophages. Moreover, the precise mechanism by which the components of RC@RMPs synergistically induce M1 macrophage polarization is not yet fully characterized. Further efforts should seek to screen novel genes that specifically contribute to the ferroptosis of prostate cancer cells and elucidate the relationship between macrophage polarization, changes in mitochondrial functionality, and ferroptosis, to support the development of more efficient and accurate therapies for prostate cancer.

### Supplementary Information

**Additional file1: Figure S1.** HPLC profile of RSL3 and RSL3 in RMPs. **Figure S2.** Optimization of CT20p to RMPs ratio. The electroporation parameters were set as 500V voltage, 125 μF capacitance, and exponential decay wave mode. A set mass of 100 μg RMPs was tested. FITC-conjugated CT20p was measured through relative fluorescence units (RFU) using a microplate reader. P-values were calculated by one-way analysis of variance (ANOVA). ***P < 0.001, ns stands for no significant difference. **Figure S3.** Release profile of RSL3 and CT20p. RC@RMPs were incubated with mice serum for different times at 37 °C and collected for measurement of RSL3 (A) and CT20p (B) concentrations. **Figure S4.** Evaluation of cellular uptake mechanisms of RC@RMPs. (A) RM-1 cells were pre-incubated with various inhibitors for 2 hours, washed, then further incubated with DiO-labelled RC@RMPs for 4 hours. The MFI in the FL1 channel of RM-1 cells was determined by flow cytometry. (B) RM-1 cells were incubated with DiO-labelled RC@RMPs for 24 hours, washed, then further stained with Hoechst 33324 and Lysotracker. Scale bar: 20 μm. P-values were calculated by one-way analysis of variance (ANOVA). **P < 0.01, ***P < 0.001 and ****P < 0.0001. **Figure S5.** Effect of RSL3 and CT20p loading on Calreticulin (CRT) expression levels in RM-1 cells. RM-1 cells were incubated with RMPs, RSL3@RMPs, CT20p@RMPs, or RC@RMPs for 24 hours. The level of CRT in RM-1 lysates was determined by western blotting. GAPDH was used as the loading control. **Figure S6.** The combination of RSL3 and CT20p synergizes to produce lipid ROS in RM-1 cells. RM-1 cells were labelled with C11B-BODIPY 581/591 (10 μM) in 1 mL PBS for 30 min at 37 °C and washed with PBS twice. Then the cells were resuspended in 200 μL PBS and analyzed via flow cytometry. P-values were calculated by one-way analysis of variance (ANOVA). **P < 0.01, ***P < 0.001 and ****P < 0.0001. **Figure S7.** DCs and macrophages ingest RMPs and RC@RMPs in a dose-dependent manner. (A) DC2.4 and (B) RAW264.7 cells were incubated with DiO-labelled RMPs and RC@RMPs for 24 hours. MFI in the FL1 channel was measured by flow cytometry. P-values were calculated by one-way analysis of variance (ANOVA). *P < 0.05, **P < 0.01 and ****P < 0.0001. **Figure S8.** Toxicity assessment of all the RMPs on DCs and macrophages. DC2.4 (A) and RAW264.7 cells (B) were incubated with RMPs, RSL3@RMPs, CT20p@RMPs, or RC@RMPs for 24 h. Relative cell growth of RM-1 cells was measured by CCK-8 assay. **Figure S9.** RC@RMPs reprogram macrophage polarization. RAW264.7 cells were cultured in 24-well plates (30,000 cells per well) and then incubated with PBS (control), RMPs, RSL3@RMPs, CT20p@RMPs, or RC@RMPs for 24 h. To analyze the polarization of macrophages, the expression levels of CD86 (A) and CD206 (B and C) in RAW264.7 cells were measured. P-values were calculated by one-way analysis of variance (ANOVA). ****P < 0.0001. **Figure S10.** RC@RMPs enhance ROS production in DCs. DC2.4 cells were cultured in 24-well plates (30,000 cells per well) and then incubated with PBS (control), RMPs, RSL3@RMPs, CT20p@RMPs, or RC@RMPs for 24 hours. ROS levels were determined by quantifying H2DCFDA probe fluorescence by flow cytometry. P-values were calculated by one-way analysis of variance (ANOVA). *P < 0.05, **P < 0.01 and ****P < 0.0001. **Figure S11.** Assessment of the ability of RC@RMPs to activate the cGAS-STING pathway. DC2.4 cells were incubated with RMPs, RSL3@RMPs, CT20p@RMPs, or RC@RMPs for 24 h. The levels of p-STING, p-NFκB, STING, and NF-κB in DC2.4 lysates were analyzed by Western blotting. GAPDH and Laminin B1 were used as loading controls for cytoplasmic and nuclear proteins, respectively. **Figure S12.** RC@RMPs upregulate the level of ROS and Fe2+ in macrophages. RAW264.7 cells were treated as per Figure S9. ROS and Fe2+ levels were measured by H2DCFDA probe and PGSK signal, respectively, as quantified by flow cytometry. P-values were calculated by one-way analysis of variance (ANOVA). *P < 0.05, **P < 0.01, ***P < 0.001 and ****P < 0.0001. **Figure S13.** Distribution of RMPs and RC@RMPs in vivo. 100 μg of PKH26-labelled RMPs and RC@RMPs were intratumorally injected and the mice were sacrificed at 24 hours after treatment. (A) Images of key organs and tumors acquired using the IVIS system. (B) Representative immunofluorescence images showing colocalization of RMPs and Ly6G+ neutrophils in tumor tissue at 24 h after treatment. Scale bar (original image): 200 μm, Scale bar (zoomed-in image): 50 μm. **Figure S14.** Representative flow cytometry pseudocolor plots of data shown in Figure 4A. **Figure S15.** Tumor size measurements prior to RMPs treatments. RM-1 tumor cells (1×106 cells in 100 μL PBS) were subcutaneously implanted into the right back. Seven days after tumor inoculation, tumor volumes were calculated by the formula V = (L × W2) / 2. **Figure S16.** The flow cytometry gating strategy for different immune cells in Figure 5D-L. **Figure S17.** Representative flow cytometry pseudocolor plots of data shown in Figure 5D-L. **Figure S18.** The cytokine spectrum is changed by RC@RMPs and anti-PD-1 mAb treatment. The levels of related cytokines in the homogenates of RM-1 tumors in mice treated according to Figure 5A were measured using the Cytokine Release Syndrome Panel. P-values were calculated by one-way analysis of variance (ANOVA). *P < 0.05, **P < 0.01, ***P < 0.001 and ****P < 0.0001, ns stands for no significance. **Figure S19.** Assessment of immune cells infiltration levels in tumor tissues. The RM-1 tumor-bearing mice were treated and sacrificed according to Figure 5A. Representative immunofluorescence images showing the number of CD3+ T cells (A) and Ly6G+ neutrophils (B) in tumor tissue, Scale bar (original image): 200 μm. Scale bar (zoomed-in image): 50 μm. **Figure S20.** Confirmation of immune cell subset depletion. The percentages of CD4+ T cells, CD8+ T cells, neutrophils, and macrophages in peripheral blood were determined by flow cytometry 24 hours after treatment with the corresponding agents for immunocyte depletion. **Figure S21.** In vivo toxicity assessment. (A) The levels of alanine transaminase (ALT), aspartate transaminase (AST), blood urea nitrogen (BUN), and creatinine (CREA) in serum for the evaluation of hepatic and renal function. (B) Histopathological examination of heart, liver, spleen, lung, and kidney. Scale bar: 200 μm. Table 1 Complete Blood Count (CBC) of the mice after treatment with RMPs.

## Data Availability

The authors declare that all data supporting the results of this study are available in the paper and supplementary information. Source data are provided in this paper.

## Abbreviations

CRPC: Castration-resistant prostate cancer
ICD: Immunogenic cell death
TME: Tumor microenvironment
DAMPs: Damage-associated molecular patterns
RMPs: Radiated tumor cell-derived microparticles
CT20p: CT20 peptide
RC@RMPs: RSL-3- and CT20p-loaded RMPs
DC: Dendritic cell
MDSCs: Myeloid-derived suppressor cells
HPLC: High performance liquid chromatography
dLNs: Draining lymph nodes
TEM: Transmission electron microscopy
MFI: Mean fluorescence intensity
APCs: Antigen presenting cells
TAMs: Tumor-associated macrophages
CVME: Caveolin-mediated endocytosis
ALT: Alanine transaminase
AST: Aspartate transaminase
BUN: Blood urea nitrogen
CREA: Creatinine
CBC: Complete blood count
P-TEFb: Positive transcription elongation factor b
ICT: Immune checkpoint therapy
