# Supplementary material for: Irradiated microparticles suppress prostate cancer by tumor microenvironment reprogramming and ferroptosis
Source: J Nanobiotechnology. 2024 May 5;22:225. doi: 10.1186/s12951-024-02496-3 (PMC11070086; doi:10.1186/s12951-024-02496-3)
Supplement: Supplementary file 1 — Additional file1: Figure S1. HPLC profile of RSL3 and RSL3 in RMPs. Figure S2. Optimization of CT20p to RMPs ratio. The electroporation parameters were set as 500V voltage, 125 μF capacitance, and exponential decay wave mode. A set mass of 100 μg RMPs was tested. FITC-conjugated CT20p was measured through relative fluorescence units (RFU) using a microplate reader. P-values were calculated by one-way analysis of variance (ANOVA). ***P < 0.001, ns stands for no significant difference. Figure S3. Release profile of RSL3 and CT20p. RC@RMPs were incubated with mice serum for different times at 37 °C and collected for measurement of RSL3 (A) and CT20p (B) concentrations. Figure S4. Evaluation of cellular uptake mechanisms of RC@RMPs. (A) RM-1 cells were pre-incubated with various inhibitors for 2 hours, washed, then further incubated with DiO-labelled RC@RMPs for 4 hours. The MFI in the FL1 channel of RM-1 cells was determined by flow cytometry. (B) RM-1 cells were incubated with DiO-labelled RC@RMPs for 24 hours, washed, then further stained with Hoechst 33324 and Lysotracker. Scale bar: 20 μm. P-values were calculated by one-way analysis of variance (ANOVA). **P < 0.01, ***P < 0.001 and ****P < 0.0001. Figure S5. Effect of RSL3 and CT20p loading on Calreticulin (CRT) expression levels in RM-1 cells. RM-1 cells were incubated with RMPs, RSL3@RMPs, CT20p@RMPs, or RC@RMPs for 24 hours. The level of CRT in RM-1 lysates was determined by western blotting. GAPDH was used as the loading control. Figure S6. The combination of RSL3 and CT20p synergizes to produce lipid ROS in RM-1 cells. RM-1 cells were labelled with C11B-BODIPY 581/591 (10 μM) in 1 mL PBS for 30 min at 37 °C and washed with PBS twice. Then the cells were resuspended in 200 μL PBS and analyzed via flow cytometry. P-values were calculated by one-way analysis of variance (ANOVA). **P < 0.01, ***P < 0.001 and ****P < 0.0001. Figure S7. DCs and macrophages ingest RMPs and RC@RMPs in a dose-dependent manner. (A) DC2 [file 12951_2024_2496_MOESM1_ESM.docx]

**Supporting information**

**Irradiated Microparticles for Treatment of Prostate cancer by Reprograming Tumor Microenvironment and Inducing Multi-modal Death of Tumor**

Zihan Deng^a,1^, Binghui Li^b,c,1^, Muyang Yang^d,1^, Lisen Lu^d^, Jonathan F. Lovell^e^, Xiantao Zeng^b,c,^*, Weidong Hu^a,^*, Honglin Jin^d,^*

^a^Department of Thoracic Surgery, ZhongNan Hospital of Wuhan University, Wuhan, Hubei, China

^b^Center for Evidence-Based and Translational Medicine, Zhongnan Hospital of Wuhan University, Wuhan, China

^c^Department of Urology, Zhongnan Hospital of Wuhan University, Wuhan, China

^d^College of Biomedicine and Health and College of Life Science and Technology, Huazhong Agricultural University, Wuhan, 430070, China

^e^Department of Biomedical Engineering, University at Buffalo, State University of New York, Buffalo, NY 14260, USA

^1^ These authors contributed equally to this work.

*Corresponding authors.

E-mail addresses: [jin@hust.edu.cn](mailto:jin@hust.edu.cn) (H. Jin), [huwd@whu.edu.cn](mailto:huwd@whu.edu.cn) (W. Hu), [zengxiantao1128@whu.edu.cn](mailto:zengxiantao1128@whu.edu.cn) (X. Zeng)


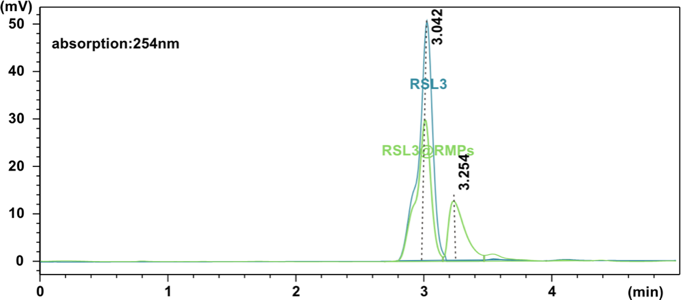


**Figure S1. HPLC profile of RSL3 and RSL3 in RMPs.**

**
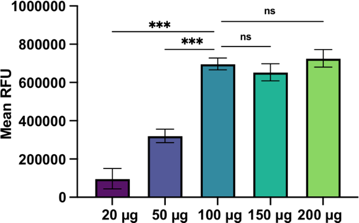
**

**Figure S2. Optimization of CT20p to RMPs ratio**. The electroporation parameters were set as 500V voltage, 125 μF capacitance, and exponential decay wave mode. A set mass of 100 μg RMPs was tested. FITC-conjugated CT20p was measured through relative fluorescence units (RFU) using a microplate reader. P-values were calculated by one-way analysis of variance (ANOVA). ****P* < 0.001, ns stands for no significant difference.

**
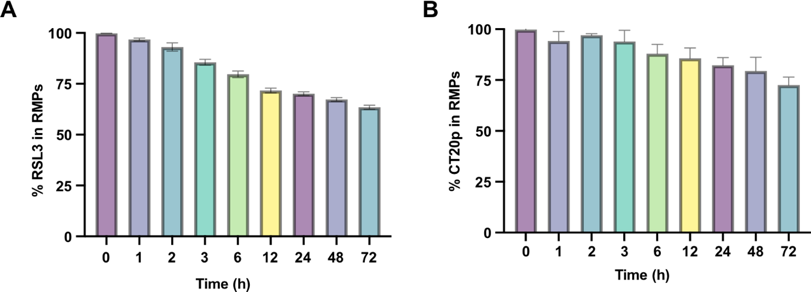
**

**Figure S3. Release profile of RSL3 and CT20p.** RC@RMPs were incubated with mice serum for different times at 37 °C and collected for measurement of RSL3 (A) and CT20p (B) concentrations.

**
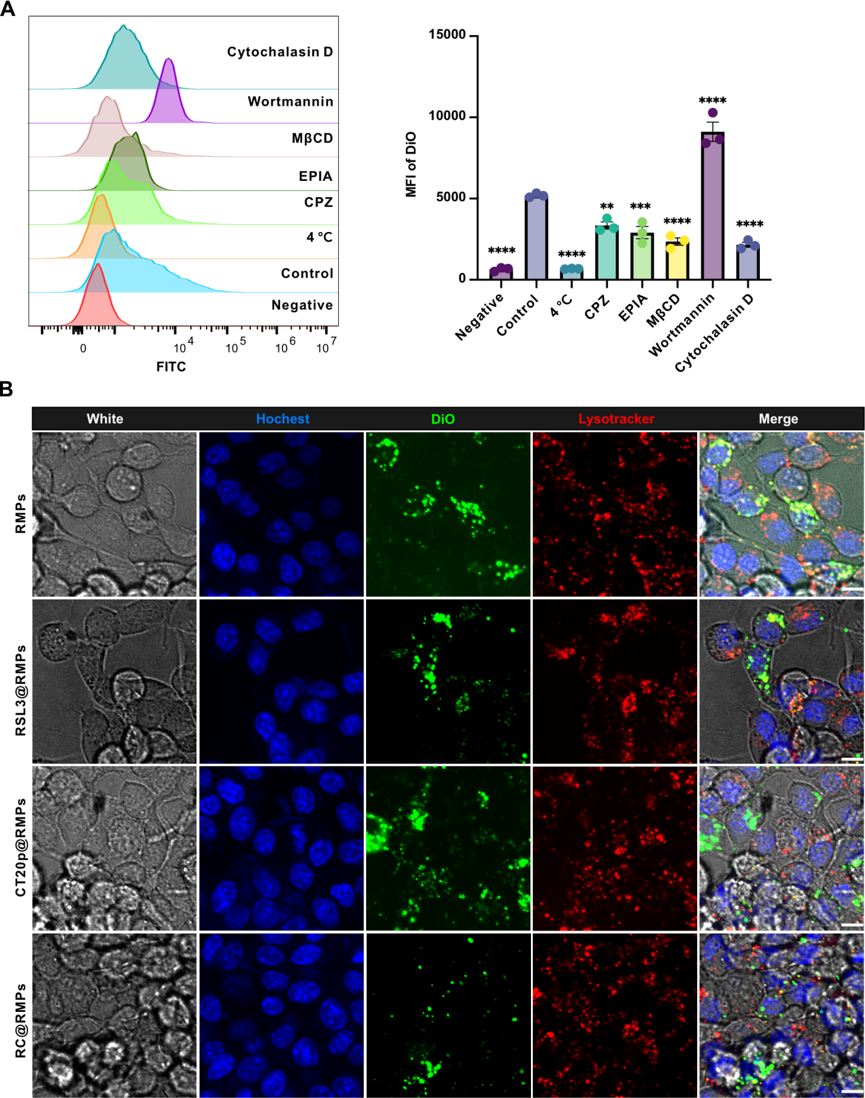
**

**Figure S4.** **Evaluation of cellular uptake mechanisms of RC@RMPs**. (A) RM-1 cells were pre-incubated with various inhibitors for 2 hours, washed, then further incubated with DiO-labelled RC@RMPs for 4 hours. The MFI in the FL1 channel of RM-1 cells was determined by flow cytometry. (B) RM-1 cells were incubated with DiO-labelled RC@RMPs for 24 hours, washed, then further stained with Hoechst 33324 and Lysotracker. Scale bar: 20 μm. P-values were calculated by one-way analysis of variance (ANOVA). ***P* < 0.01, ****P* < 0.001 and *****P* < 0.0001.

**
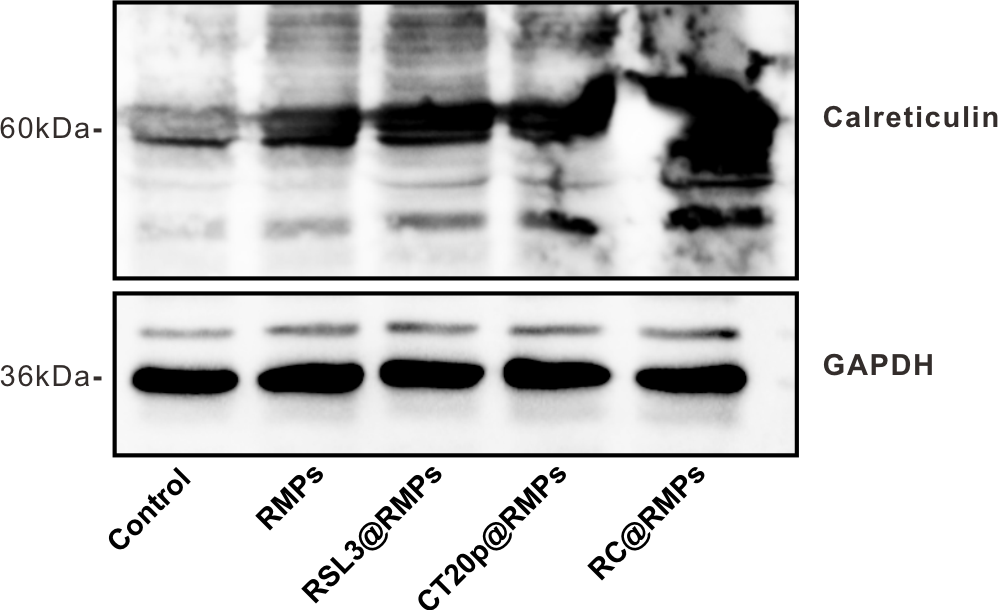
**

**Figure S5. Effect of RSL3 and CT20p loading on Calreticulin (CRT) expression levels in RM-1 cells.** RM-1 cells were incubated with RMPs, RSL3@RMPs, CT20p@RMPs, or RC@RMPs for 24 hours. The level of CRT in RM-1 lysates was determined by western blotting. GAPDH was used as the loading control.


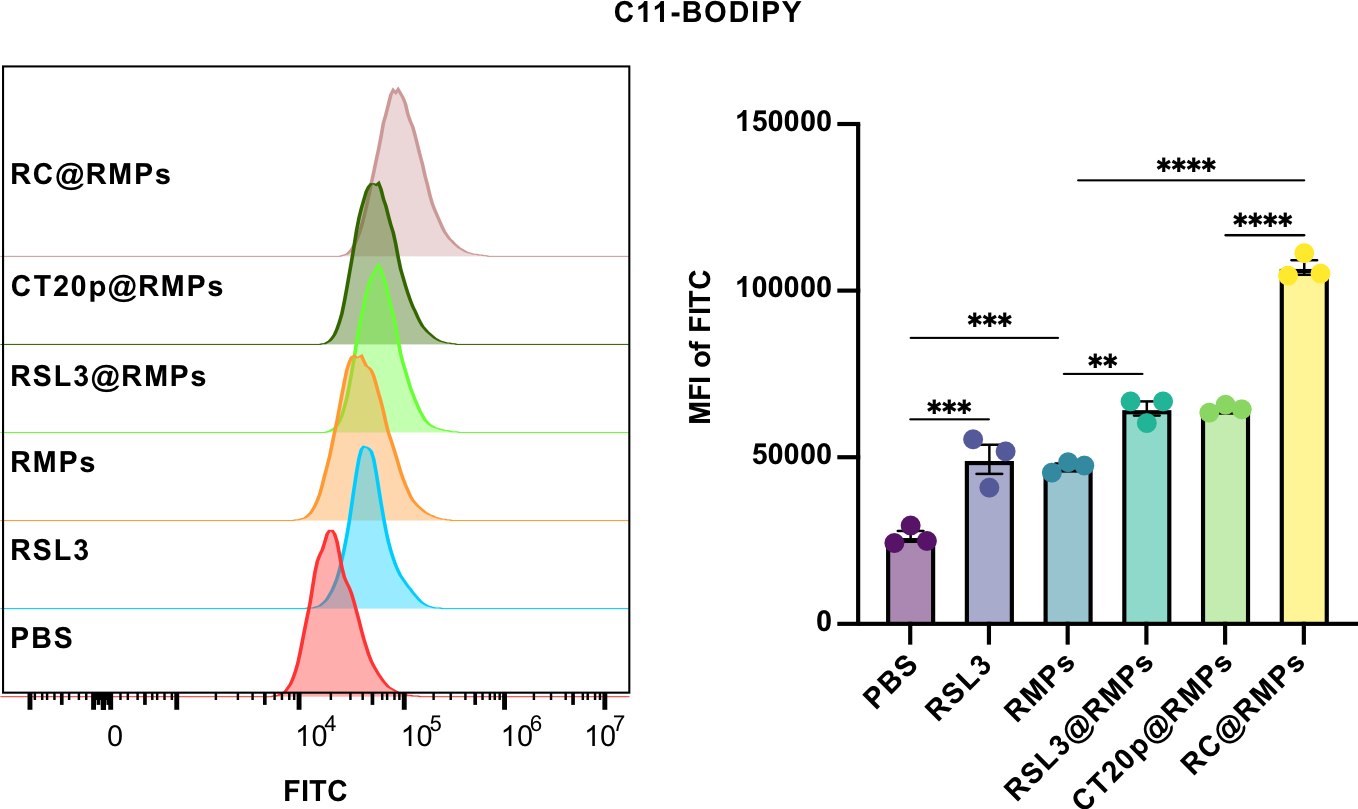


**Figure S6. The combination of RSL3 and CT20p synergizes to produce lipid ROS in RM-1 cells.** RM-1 cells were labelled with C11B-BODIPY 581/591 (10 μM) in 1 mL PBS for 30 min at 37 °C and washed with PBS twice. Then the cells were resuspended in 200 μL PBS and analyzed *via* flow cytometry. P-values were calculated by one-way analysis of variance (ANOVA). ***P* < 0.01, ****P* < 0.001 and *****P* < 0.0001.


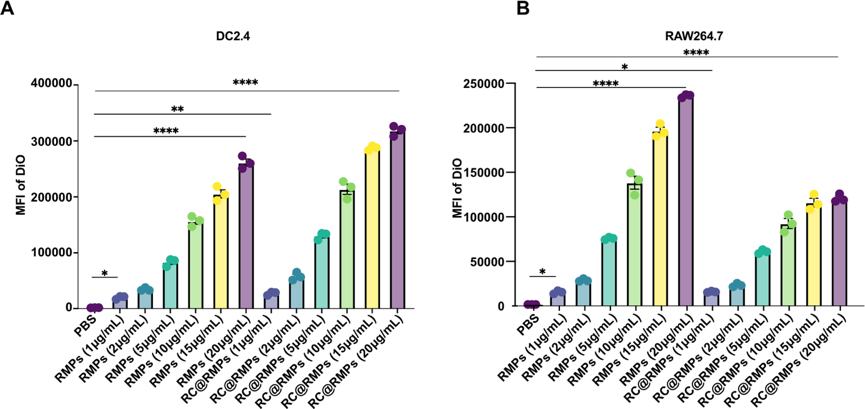


**Figure S7. DCs and macrophages ingest RMPs and RC@RMPs in a dose-dependent manner.** (A) DC2.4 and (B) RAW264.7 cells were incubated with DiO-labelled RMPs and RC@RMPs for 24 hours. MFI in the FL1 channel was measured by flow cytometry. P-values were calculated by one-way analysis of variance (ANOVA). **P* < 0.05, ***P* < 0.01 and *****P* < 0.0001.


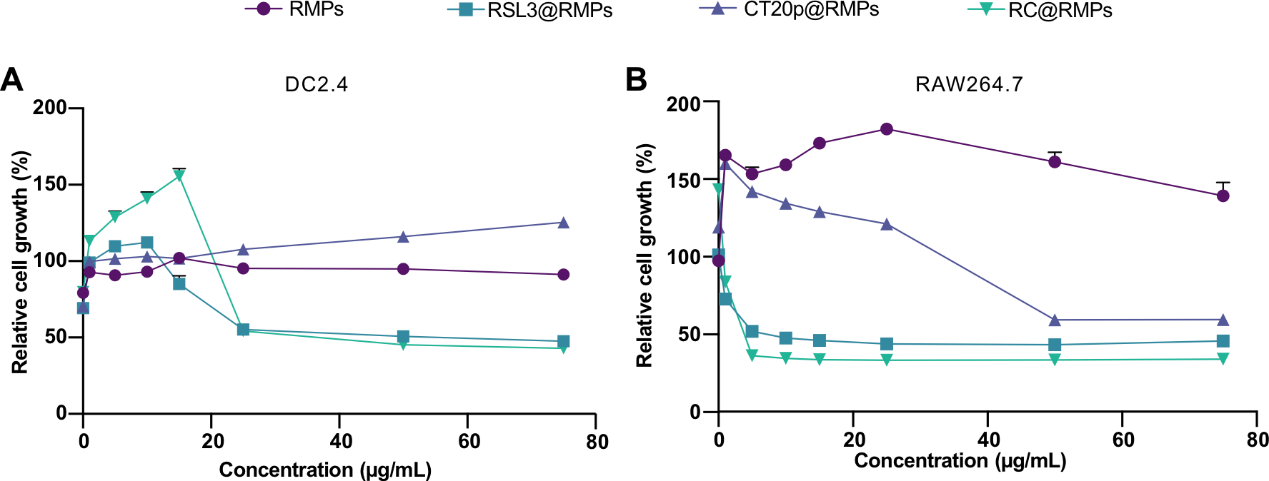


**Figure S8. Toxicity assessment of all the RMPs on DCs and macrophages.** DC2.4 (A) and RAW264.7 cells (B) were incubated with RMPs, RSL3@RMPs, CT20p@RMPs, or RC@RMPs for 24 hours. Relative cell growth of RM-1 cells was measured by CCK-8 assay.

**
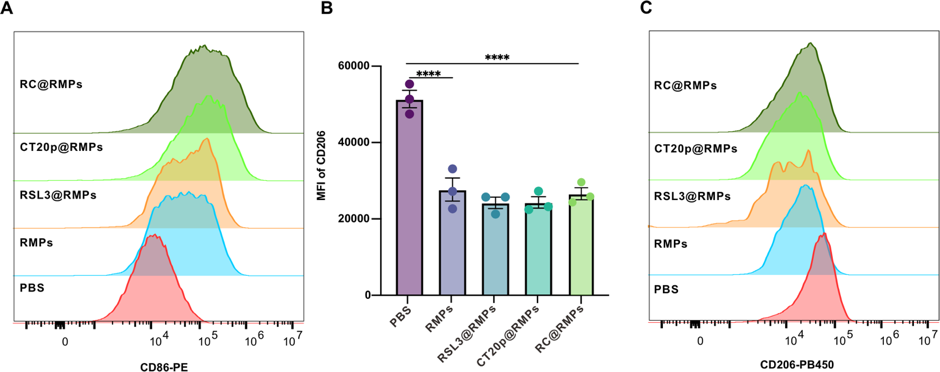
**

**Figure S9. RC@RMPs reprogram macrophage polarization.** RAW264.7 cells were cultured in 24-well plates (30,000 cells per well) and then incubated with PBS (control), RMPs, RSL3@RMPs, CT20p@RMPs, or RC@RMPs for 24 hours. To analyze the polarization of macrophages, the expression levels of CD86 (A) and CD206 (B and C) in RAW264.7 cells were measured. P-values were calculated by one-way analysis of variance (ANOVA). *****P* < 0.0001.


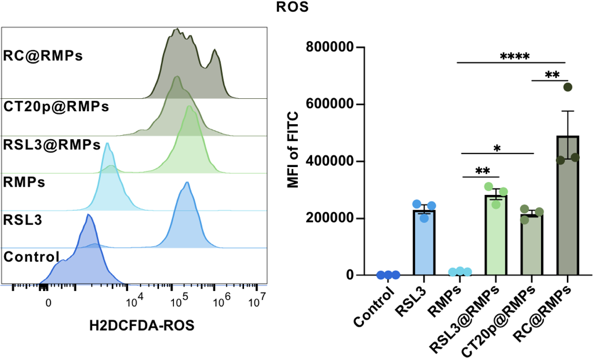


**Figure S10. RC@RMPs enhance ROS production in DCs**. DC2.4 cells were cultured in 24-well plates (30,000 cells per well) and then incubated with PBS (control), RMPs, RSL3@RMPs, CT20p@RMPs, or RC@RMPs for 24 hours. ROS levels were determined by quantifying H2DCFDA probe fluorescence by flow cytometry. P-values were calculated by one-way analysis of variance (ANOVA). **P* < 0.05, ***P* < 0.01 and *****P* < 0.0001.


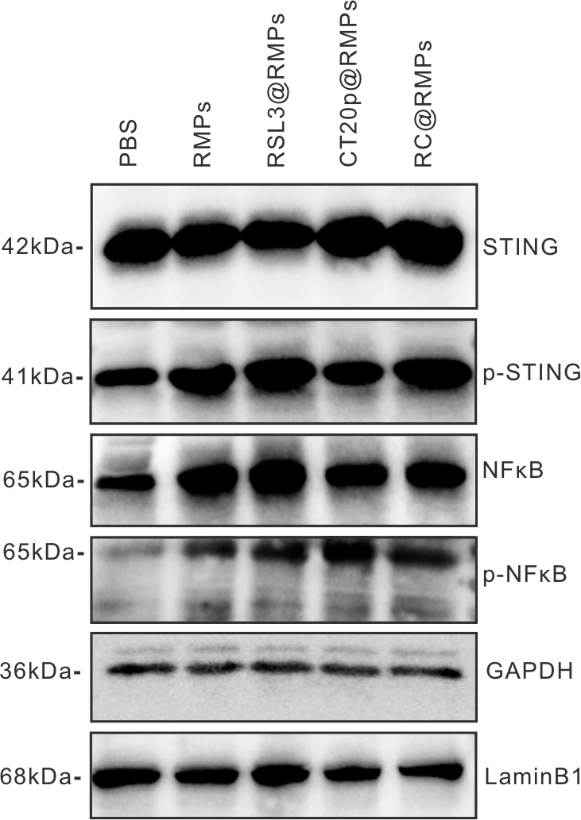


**Figure S11. Assessment of the ability of RC@RMPs to activate the cGAS-STING pathway.** DC2.4 cells were incubated with RMPs, RSL3@RMPs, CT20p@RMPs, or RC@RMPs for 24 hours. The levels of p-STING, p-NFκB, STING, and NF-κB in DC2.4 lysates were analyzed by Western blotting. GAPDH and Laminin B1 were used as loading controls for cytoplasmic and nuclear proteins, respectively.

**
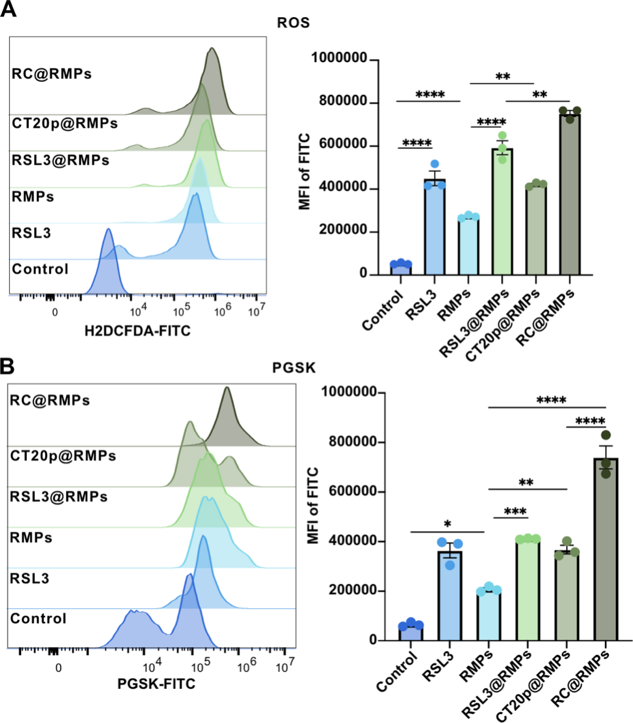
**

**Figure S12. RC@RMPs upregulate the level of ROS and Fe^2+^ in macrophages.** RAW264.7 cells were treated as per Figure S9. ROS and Fe^2+^ levels were measured by H2DCFDA probe and PGSK signal, respectively, as quantified by flow cytometry. P-values were calculated by one-way analysis of variance (ANOVA). **P* < 0.05, ***P* < 0.01, ****P* < 0.001 and *****P* < 0.0001.


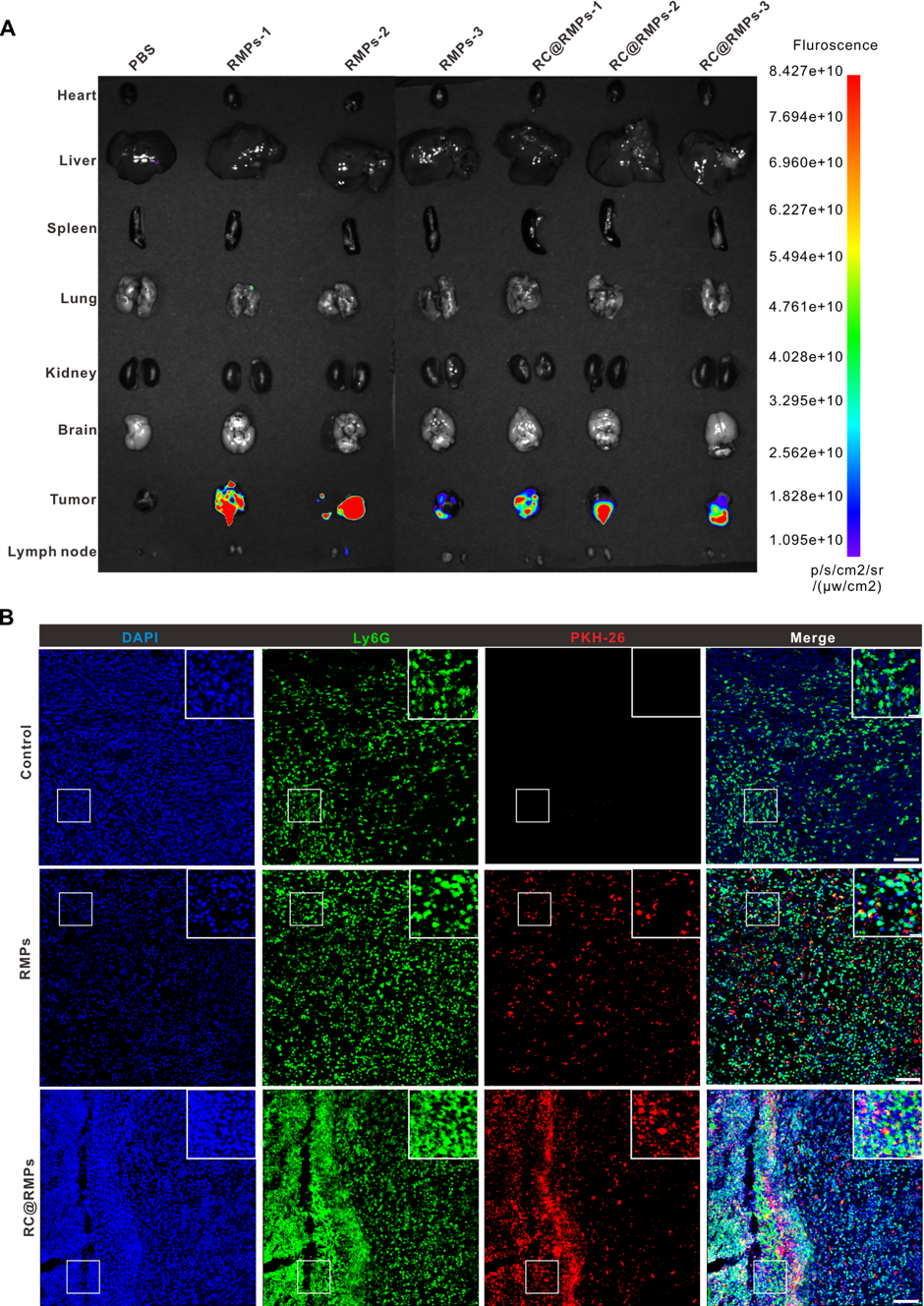


**Figure S13. Distribution of RMPs and RC@RMPs *in vivo*.** 100 μg of PKH26-labelled RMPs and RC@RMPs were intratumorally injected and the mice were sacrificed at 24 hours after treatment. (A) Images of key organs and tumors acquired using the IVIS system. (B) Representative immunofluorescence images showing colocalization of RMPs and Ly6G^+^ neutrophils in tumor tissue at 24 hours after treatment. Scale bar (original image): 200 μm, Scale bar (zoomed-in image): 50 μm.

**
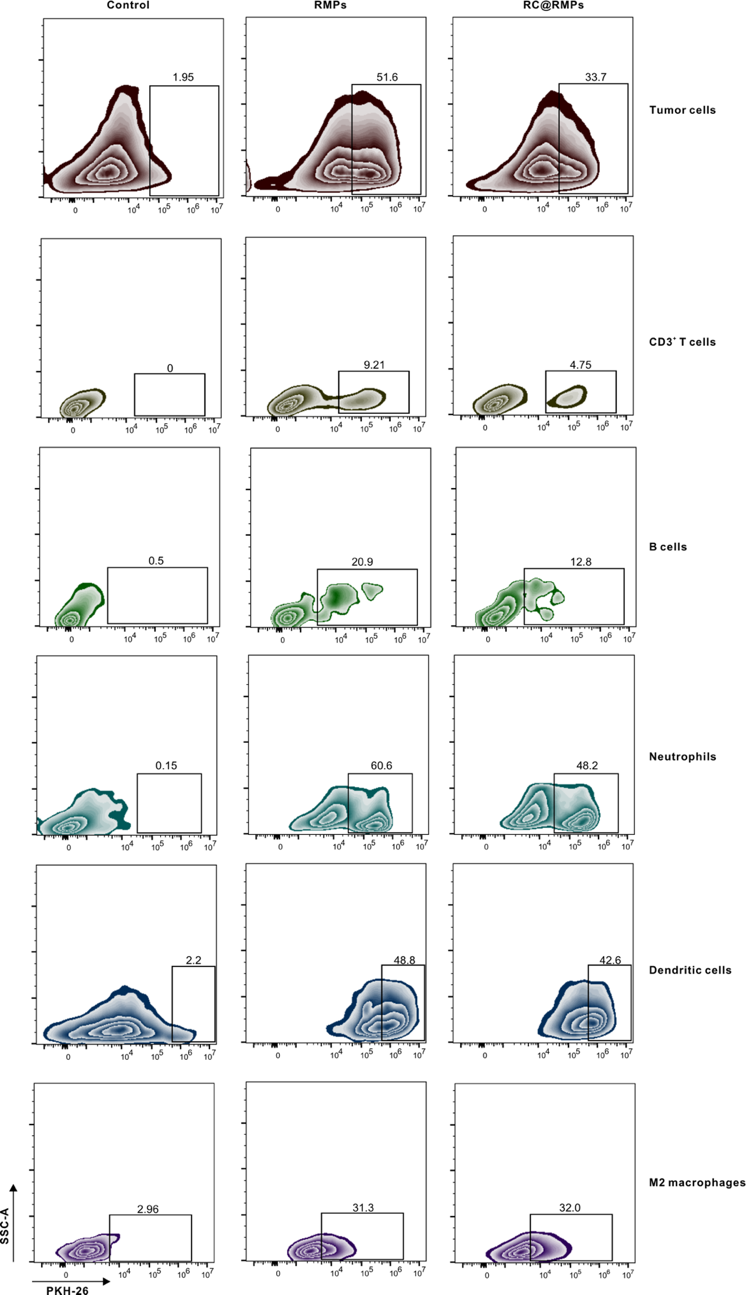
**

**Figure S14. Representative flow cytometry pseudocolor plots of data shown in Figure 4A.**

**
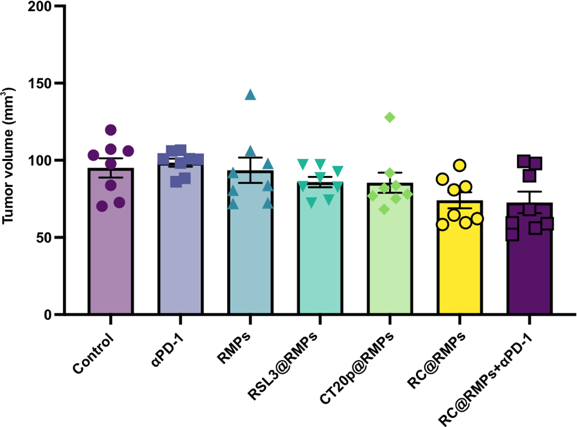
**

**Figure S15. Tumor size measurements prior to RMPs treatments.** RM-1 tumor cells (1×10^6^ cells in 100 μL PBS) were subcutaneously implanted into the right back. Seven days after tumor inoculation, tumor volumes were calculated by the formula *V* = (*L* × *W^2^*) / 2.


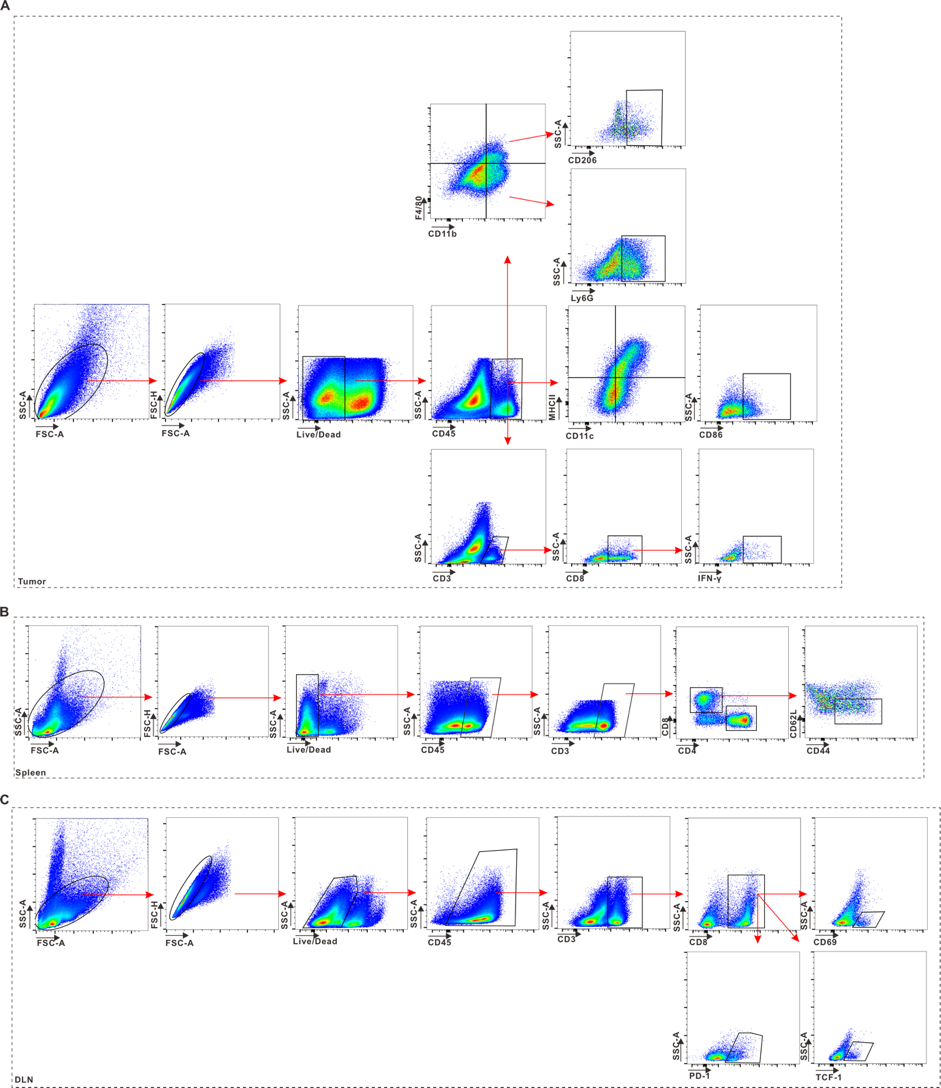


**Figure S16. The flow cytometry gating strategy for different immune cells in Figure 5D-L.**

**
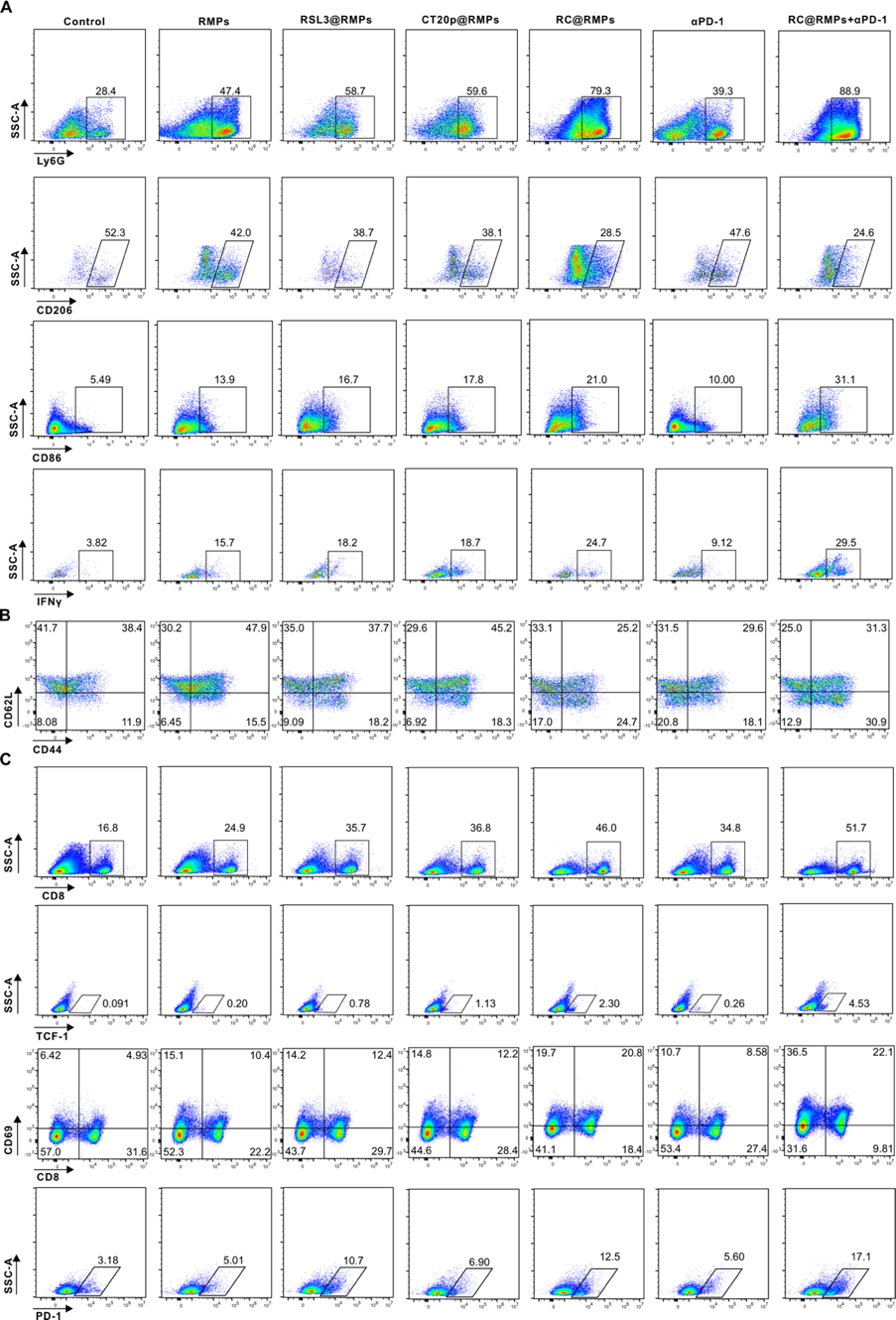
**

**Figure S17. Representative flow cytometry pseudocolor plots of data shown in Figure 5D-L.**


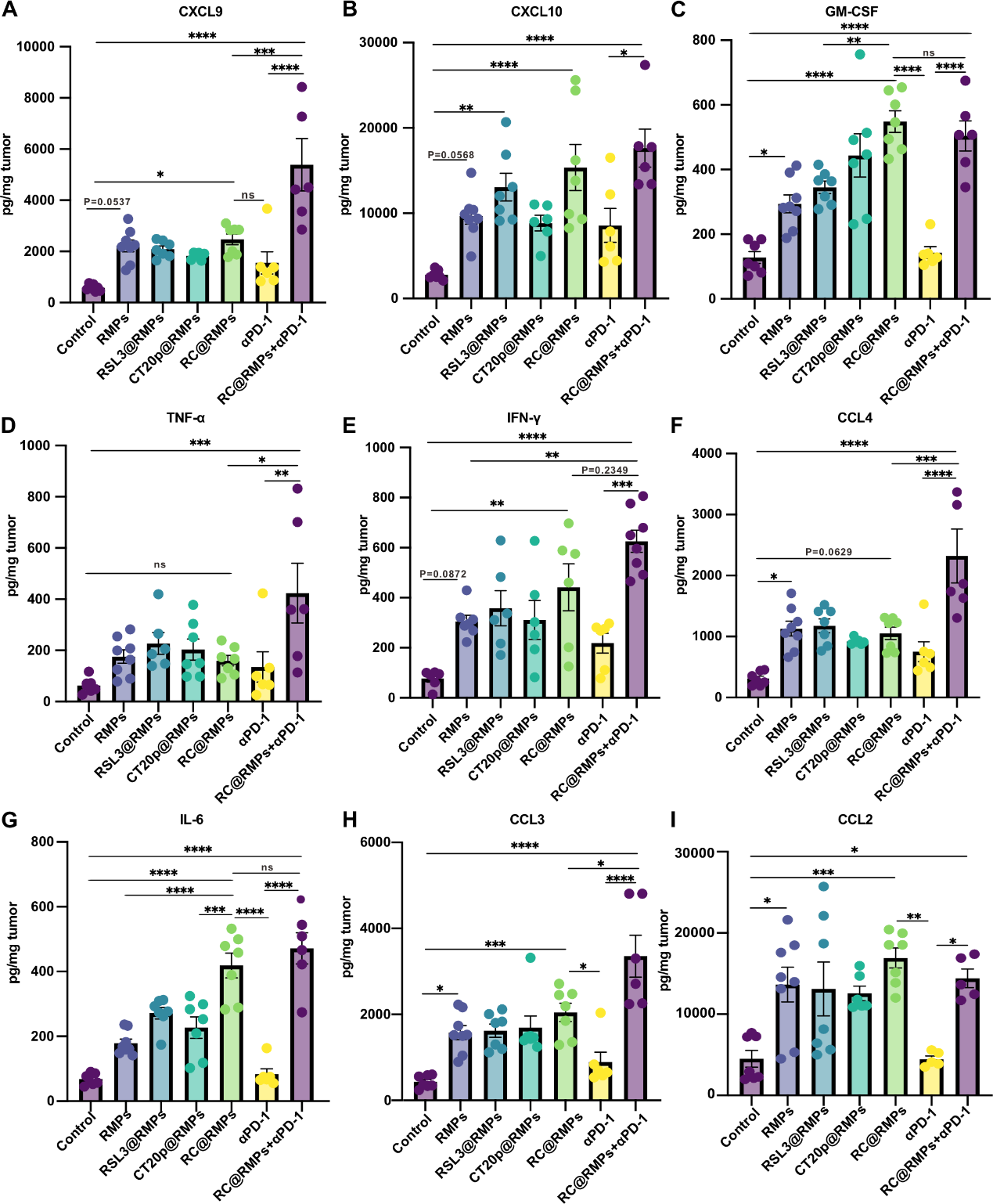


**Figure S18. The cytokine spectrum is changed by RC@RMPs and anti-PD-1 mAb treatment.** The levels of related cytokines in the homogenates of RM-1 tumors in mice treated according to Figure 5A were measured using the Cytokine Release Syndrome Panel. P-values were calculated by one-way analysis of variance (ANOVA). **P* < 0.05, ***P* < 0.01, ****P* < 0.001 and *****P* < 0.0001, ns stands for no significance.


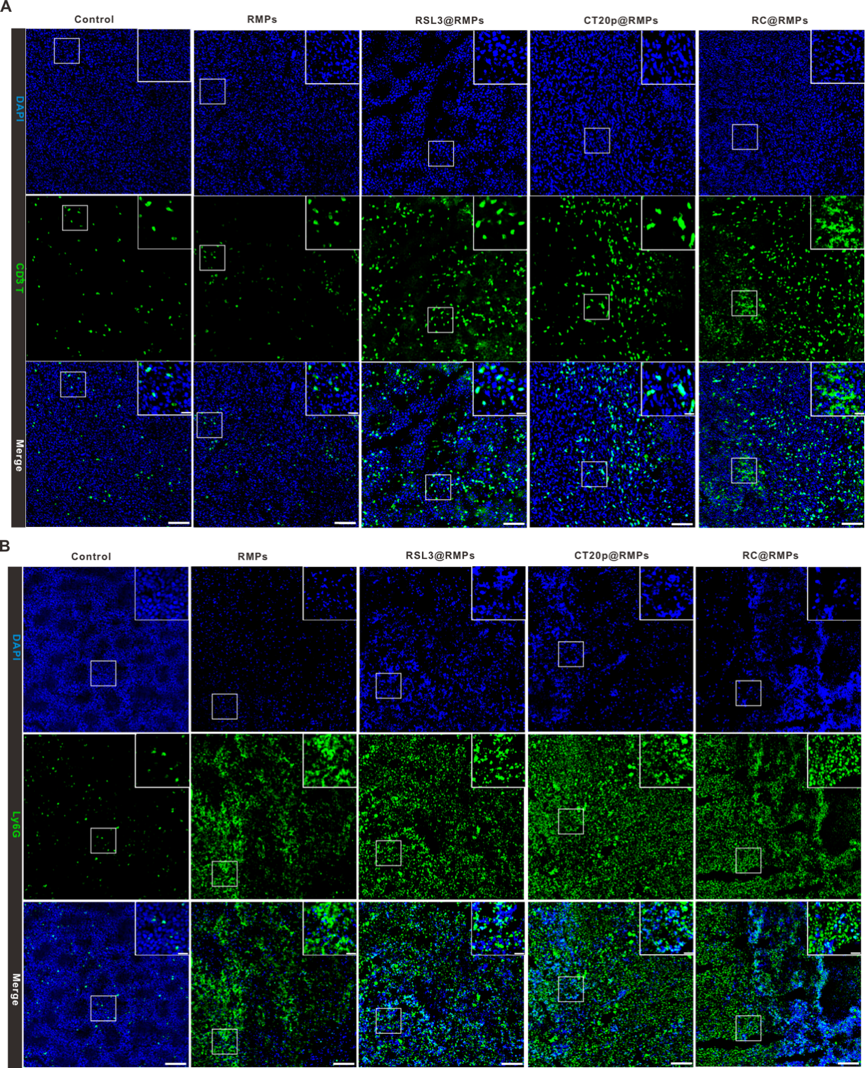


**Figure S19. Assessment of immune cells infiltration levels in tumor tissues.** The RM-1 tumor-bearing mice were treated and sacrificed according to Figure 5A. Representative immunofluorescence images showing the number of CD3^+^ T cells (A) and Ly6G^+^ neutrophils (B) in tumor tissue, Scale bar (original image): 200 μm. Scale bar (zoomed-in image): 50 μm.


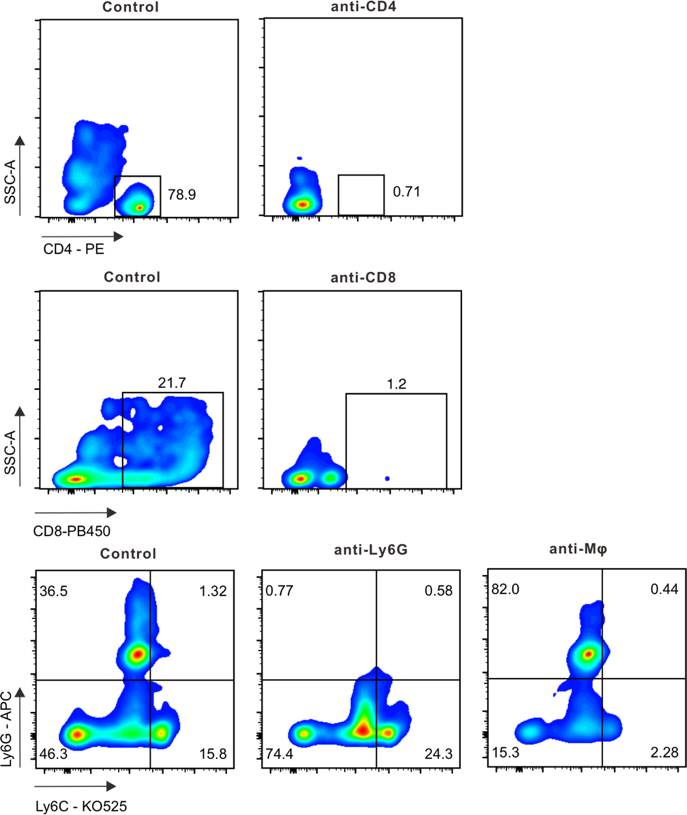


**Figure S20. Confirmation of immune cell subset depletion.** The percentages of CD4^+^ T cells, CD8^+^ T cells, neutrophils, and macrophages in peripheral blood were determined by flow cytometry 24 hours after treatment with the corresponding agents for immunocyte depletion.


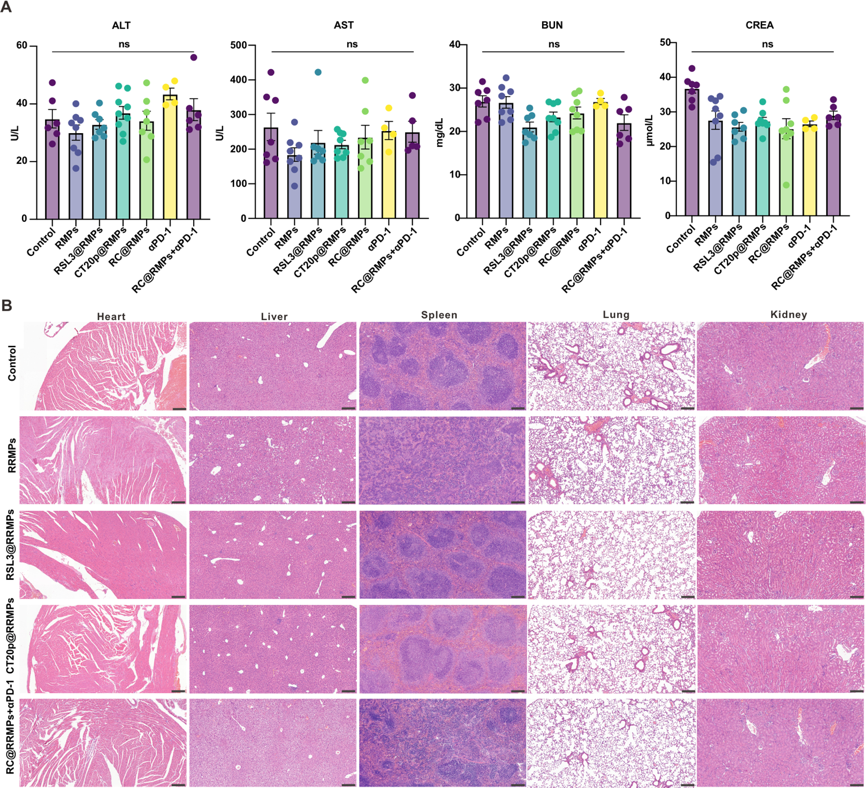


**Figure S21. *In vivo* toxicity assessment.** (A) The levels of alanine transaminase (ALT), aspartate transaminase (AST), blood urea nitrogen (BUN), and creatinine (CREA) in serum for the evaluation of hepatic and renal function. (B) Histopathological examination of heart, liver, spleen, lung, and kidney. Scale bar: 200 μm.

**Table 1 Complete Blood Count (CBC) of the mice after treatment with RMPs.**

|  | Control | RMPs | RSL3@RMPs | CT20p@RMPs | RC@RMPs |
| --- | --- | --- | --- | --- | --- |
| WBC | 7.7±2.4 | 8.9±1.6 | 5.8±1.3 | 8.3±2.6 | 7.8±2.4 |
| Lymph# | 4.6±1.5 | 5.6±1.9 | 3.1±1.4 | 6.7±3.4 | 6.2±1.9 |
| Mon# | 0.3±0.2 | 0.3±0.1 | 0.2±0.1 | 0.3±0.2 | 0.2±0.1 |
| Gran# | 2.8±0.7 | 3.0±0.5 | 2.5±0.6 | 3.1±1.9 | 1.4±0.5 |
| RBC | 7.9±0.3 | 7.9±0.4 | 7.8±0.1 | 7.9±0.8 | 9.1±0.3 |
| HGB | 118.0±3.6 | 113.3±2.9 | 101.7±17.1 | 118.0±15.7 | 130.0±6.2 |
| HCT | 38.0±0.9 | 36.1±0.9 | 32.6±4.2 | 36.9±4.9 | 41.2±1.5 |
| MCV | 48.4±1.2 | 45.7±1.3 | 42.2±3.8 | 46.4±1.7 | 45.6±0.5 |
| MCH | 14.9±0.3 | 14.3±0.4 | 13.0±1.6 | 14.7±0.5 | 14.3±0.4 |
| MCHC | 310±2.6 | 313.7±1.2 | 310.0±13.9 | 319.0±0.1 | 314.7±4.0 |
| RDW | 16.8±0.5 | 16.5±0.8 | 18.6±2.5 | 16.2±0.4 | 15.6±0.4 |
| PLT | 1455.7±131.5 | 1639.7±212.9 | 1976.3±266.1 | 1584.0±267.4 | 1368.3±282.7 |
| MPV | 5.2±0.2 | 5.4±0.4 | 5.4±0.2 | 5.5±0.5 | 5.3±0.3 |
| PDW | 15.9±0.1 | 15.9±0.1 | 15.6±0.2 | 15.8±0.1 | 15.7±0.1 |

**Abbreviations:** Gran, Granulocyte; HCT, Hematocrit; HGB, Hemoglobin; MCH, Mean Corpuscular Hemoglobin; MCHC, Mean Corpuscular Hemoglobin Concentration; MCV, Mean Corpuscular Volume; Mon, Monocytes; MPV, Mean Platelet Volume; PDW, Platelet Distribution Width; PLT, Platelet; RBC, Red Blood Cell; RDW, Red Blood Cell Distribution Width; WBC, White Blood Cell.
